# The crucial role of silver(i)-salts as additives in C–H activation reactions: overall analysis of their versatility and applicability

**DOI:** 10.1039/d3cs00328k

**Published:** 2023-09-01

**Authors:** Renato L. de Carvalho, Emilay B. T. Diogo, Simon L. Homölle, Suman Dana, Eufrânio N. da Silva Júnior, Lutz Ackermann

**Affiliations:** a Institute of Exact Sciences, Department of Chemistry, Federal University of Minas Gerais-UFMG, 31270-901 Belo Horizonte MG Brazil eufranio@ufmg.br https://www.eufraniolab.com; b Institut für Organische und Biomolekulare Chemie and Wöhler Research Institute for Sustainable Chemistry, Georg-August-Universität Göttingen, Tammannstrasse 2 37077 Göttingen Germany Lutz.Ackermann@chemie.uni-goettingen.de https://www.ackermann.chemie.uni-goettingen.de/ https://wisch.chemie.uni-goettingen.de/

## Abstract

Transition-metal catalyzed C–H activation reactions have been proven to be useful methodologies for the assembly of synthetically meaningful molecules. This approach bears intrinsic peculiarities that are important to be studied and comprehended in order to achieve its best performance. One example is the use of additives for the *in situ* generation of catalytically active species. This strategy varies according to the type of additive and the nature of the pre-catalyst that is being used. Thus, silver(i)-salts have proven to play an important role, due to the resulting high reactivity derived from the pre-catalysts of the main transition metals used so far. While being powerful and versatile, the use of silver-based additives can raise concerns, since superstoichiometric amounts of silver(i)-salts are typically required. Therefore, it is crucial to first understand the role of silver(i) salts as additives, in order to wisely overcome this barrier and shift towards silver-free systems.

## Abstract 2

This review discusses the important role of silver(i) salts as additives in transition-metal catalyzed C–H activation, and depicts the discussion about the current shift towards Ag-free procedures, as plausible and sustainable alternatives.

## Graphical abstract

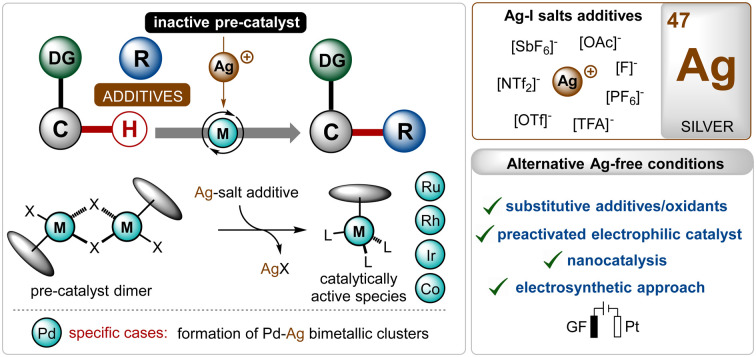

## 1. Introduction

**Scheme 1:**
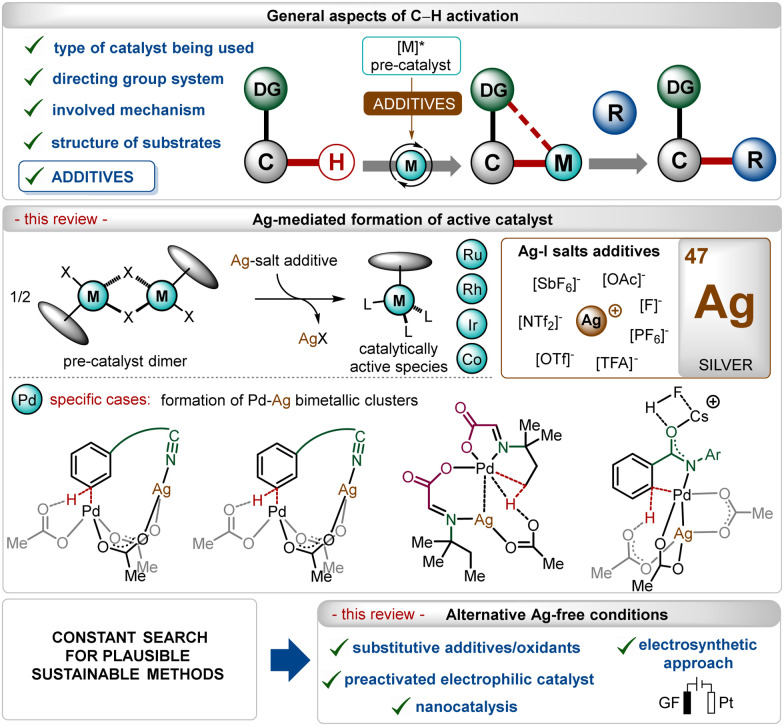
Overview.

In the past three decades, direct transition-metal catalyzed organometallic functionalization of C–H bonds, typically termed “C–H activation”, has exhibited tremendous potential in synthetic organic chemistry.^1^ This process exploits ubiquitous C–H bonds as a staple to functionalize organic molecules, enhancing the step- and atom-economies of the process. The inception and growth of C–H activation reactions facilitated the rapid construction of numerous synthetically challenging targets and thus, they have been extensively used as a potential approach for the late-stage diversification of bioactive molecules.^2^ Notably, this innovative approach somewhat depends on the nature of the applied catalyst, the use (or not) of removable directing groups, and the structure of the substrates (Scheme 1).^1,3–6^ Furthermore, specific additives play a decisive role in the process and determine the catalytic efficacy as well as turnover.^5^ In this context, the use of Ag(i)-additives is common in numerous C–H activation reactions in combination with different transition-metal catalysts, such as Ir, Rh, Ru, Co, Pd, *etc.*^1^ Even, the presence of these additives sometimes decides the fate of the transformation, making them irreplaceable in this domain.^5*b*–*d*^ These additives usually help the *in situ* generation of catalytically active species, originating usually from dimeric ruthenium-(*p*-cymene),^1*i*–*l*^ rhodium-Cp*,^1*b*,*f*^ iridium-Cp*^1*b*,*c*^ and cobalt-Cp*^1*b*^ half-sandwich pre-catalysts (Scheme 1). In contrast, for palladium catalysis, the role of the additive is shifted towards the formation of heterodimeric Pd–Ag complexes (Scheme 1).^5*b*–*d*^

In this field, among other common additives such as carboxylates, carbonates, carboxylic acids,^7^ and amine derivates,^8^ silver(i)-salts have turned out as an essential additive owing to several reasons.^5*b*–*d*,9,10^ First of all, the quick and effective interaction between the Ag(i)-cation and a halide ion (X^−^) led to the formation of AgX and this halophilicity of Ag(i)-salts stands out from other common metallic additives. Furthermore, Ag(i)-salts are recognized for their ease of forming bimetallic species bridged by carboxylate ligands, which can effectuate many transition-metal catalyzed transformations. Often, the oxidizing and Lewis-acidic properties of Ag(i)-salts also facilitate their use in C–H activation reactions.

Previously published reviews on C–H activation focused on different aspects,^4*b*–*f*^ including the nature of the directing groups,^4*g*–*j*^ transition-metal catalysis,^4*k*–*n*^ even more specifically related to 3d-metals,^4*o*–*q*^ but only a limited number rationalized the use of additives,^5*a*,7*a*^ and only fewer discussed the key role of silver(i)-based additives.^5*d*,15^ In this review, we have summarized discussions about general and specific applications of Ag(i)-salts as a pre-catalyst activator on different transition-metal catalyzed C–H activation reactions, which has not been specifically elaborated earlier. The review excludes the discussion on strategies involving Ag(i)-salts as oxidants and specifically focuses on their utility as a critical additive in the transformation. Owing to the enormous number of reports available in this regime, specific examples have been selected and analyzed. The following sections will highlight a precise overview of the role of Ag(i)-salt additives along with further discussion about the replacement of these salts with other materials and activating methods. Notably, on many occasions, an excess of inherently toxic silver(i)-salts is required to promote the transformation.^10*b*–*e*^ Thus, there is an intrinsic urge to shift the methodologies towards Ag-free conditions, improving the sustainable features of these reactions.^11^ This aspect has also been covered briefly through a precise discussion on enabling alternatives recognized to execute C–H activation reactions without Ag(i)-additives (Scheme 1).

## 2. Ruthenium catalysis with silver additives

Since the pioneering contribution by Murai and co-workers,^12^ ruthenium-catalyzed C–H activation reactions have flourished significantly in the past two decades with major contributions from Ackermann,^1*j*–*m*,13^ Dixneuf,^14^ and Miura,^15^ among others.^1*j*–*m*,16,17^

In this regime, the most used catalyst to enable C–H activation reactions is inexpensive, bench-stable, and commercially available [Ru(*p*-cymene)Cl_2_]_2_.^1*j*–*m*^ This classical half-sandwich pre-catalyst has proven to be significantly reactive in combination with large varieties of strongly as well as weakly coordinating directing groups. However, in order to obtain superior interaction with the substrates, it must be converted to its respective monomeric cationic complex. In their early findings, Ackermann validated the efficacy of the cationic Ru(ii)-complex through oxidative annulation reactions with alkynes.^13*h*,*i*^ In this context, for the *in situ* generation of the cationic Ru(ii)-complex, silver-based additives have emerged as a general candidate, which facilitate the formation of the cationic Ru(ii)-complex through the precipitation of AgCl. Generally, this process is combined with an additional carboxylate ligand, such as acetate,^1*j*–*m*^ which coordinates with the electron-deficient Ru(ii)-center with *η*^3^-coordination. The carboxylate ligand plays a crucial role in the C–H activation step through concerted metalation deprotonation (CMD) or more often a base-assisted electrophilic substitution (BIES) pathway. Notably, the electron-deficient Ru(ii)-cationic complex exhibits enhanced interactions with the weakly coordinating *O*-centered directing groups and furthers the electrophilic C–H activation strategy through decreasing the energy of LUMO of the Ru-catalyst.^18^

**Scheme 2:**
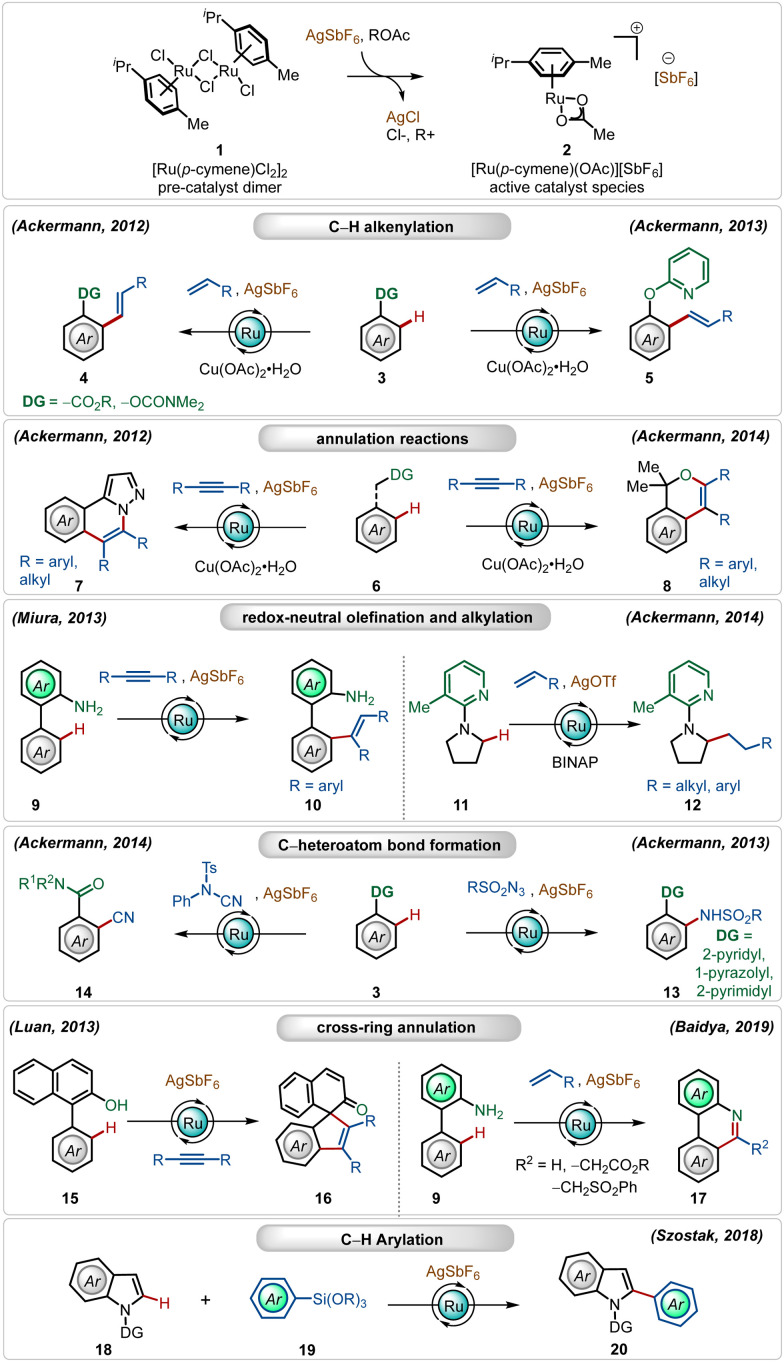
Overview of Ag-assisted [Ru]-catalyzed C–H activations.

Amongst several possible silver salts, silver hexafluoroantimonate (AgSbF_6_) is one of the commonly used additives. It has found widespread application in alkenylation,^13*j*,*h*,15*d*,19^ alkylation,^13*a*,20^ alkynylation,^21^ allylation,^22^ arylation,^23^ amidation,^24^ and annulation^13*c*,*i*,25^ reactions (Scheme 2). In this realm, the Ackermann group has a contribution, where their initial findings disclosed the oxidative olefination of carboxylic acid esters and carbamates, exploiting weakly coordinating *O*-centered directing groups.^13*j*,*h*^

Later, they also described *ortho*-C–H olefination of phenols using a removable 2-pyridyl directing group.^13*e*^ They further adopted this cationic Ru(ii)-catalyst promoted approach for oxidative annulation reactions with internal alkynes, constructing valuable heterocyclic architectures from substituted 1*H*-pyrazoles.^13*i*^ This strategy was also successful executing redox-neutral C–H activation reactions, where Miura reported an *ortho*-olefination of arenes through alkyne hydroarylation.^15*c*^

Ackermann and co-workers further found that the cationic Ru(ii)-complex could execute challenging redox-neutral alkylation of C(sp^3^)–H bonds of pyrrolidines using olefins as the alkyl source, where the AgOTf additive was found to be optimal in combination with the RuCl_2_(PPh_3_)_3_ catalyst and the BINAP ligand.^13*b*^ In this context, it is worth noting that Ru(ii)-catalyzed C(sp^3^)–H bond functionalization reactions are rare in the literature. Simultaneously, they also elaborated on challenging amidation^24^ and cyanation^13*d*^ reactions using the bench-stable Ru(ii)-catalyst along with AgSbF_6_. In all these transformations, the Ag(i)-additive played a pivotal role, the absence of which offered deteriorated outcome. Even cross-ring annulation reactions with alkynes and activated olefins^25^ and C-2 arylation of indoles^23^ were also feasible, providing modular access to a large variety of functionalized heterocycles.

**Scheme 3:**
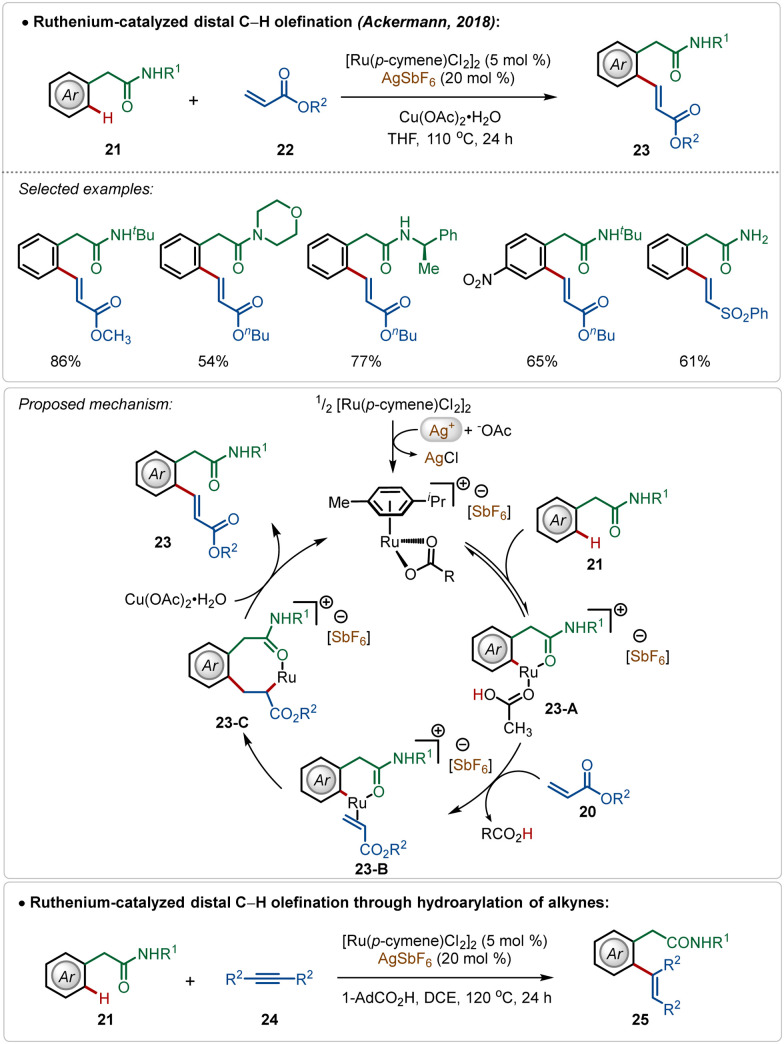
Ruthenium-catalyzed distal C–H alkenylation.

Among their other significant contribution in this domain, in 2018, the Ackermann group developed the first example of a weak coordination assisted distal C(sp^2^)–H functionalization of aryl acetamide analogues (Scheme 3).^26^ The oxidative olefination reaction tolerated diverse aryl acetamides with ease and consisted a broad scope. The reaction was executed using a cationic Ru-complex, formed *in situ* in the presence of AgSbF_6_ and the transformation failed in the absence of AgSbF_6_. The plausible mechanism involved a base-assisted internal electrophilic type substitution (BIES) pathway for the C–H activation step, forming the Ruthenacycle intermediate 23-A, which on exposure to the olefin underwent migratory insertion followed by β-hydride elimination to generate the olefinated product. The Ru(ii)-catalyst was regenerated by the Cu(ii) oxidant. Furthermore, this catalytic strategy was also applicable for redox-neutral hydroarylation of alkynes (25), albeit in this case adamantane carboxylic acid additive was necessary instead of Cu(ii)-salt.

**Scheme 4:**
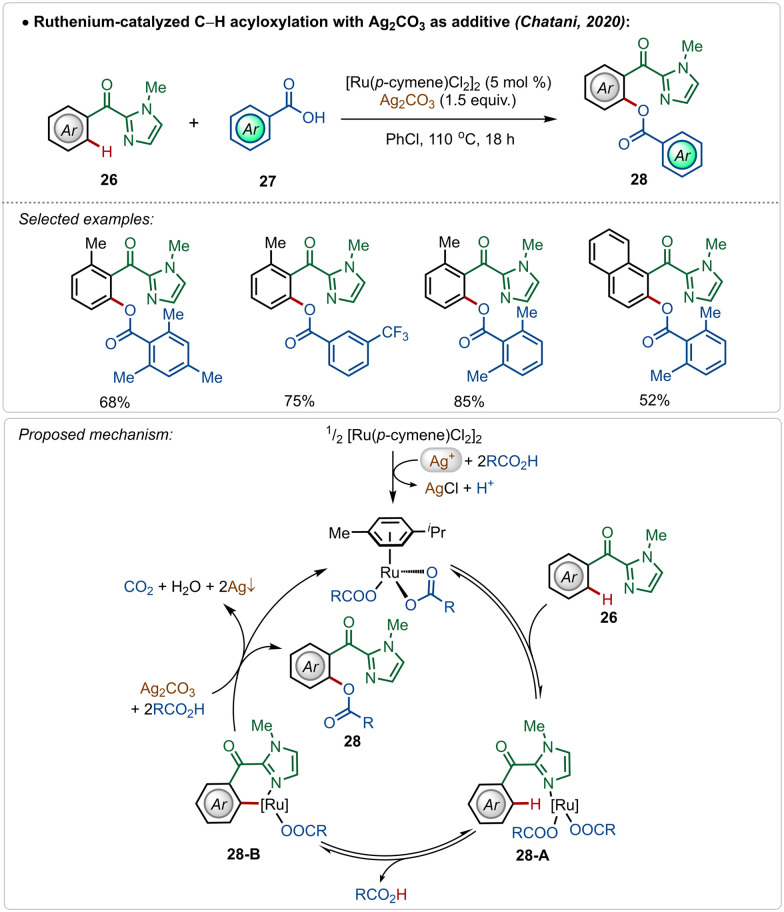
Ruthenium-catalyzed C–H acyloxylation.

In 2020, Chatani and co-workers described an interesting example of directed ruthenium-catalyzed *ortho*-C–H acyloxylation on 2-aroyl-imidazoles, using simple carboxylic acids as reaction partners (Scheme 4).^27^ Using this method, varieties of phenyl ester derivatives were synthesized in moderate to good yields. The presence of radical quencher TEMPO did not prevent the reaction, excluding the involvement of radical intermediates. Based on the plausible mechanistic proposal, the reaction starts with the usual formation of the active catalyst from the pre-catalyst in the presence of silver(i)-carbonate and carboxylic acid. The active catalyst promoted a reversible C–H activation reaction to form intermediate 28-B, which upon reductive elimination afforded the desired product along with the regeneration of the active ruthenium-catalyst.

**Scheme 5:**
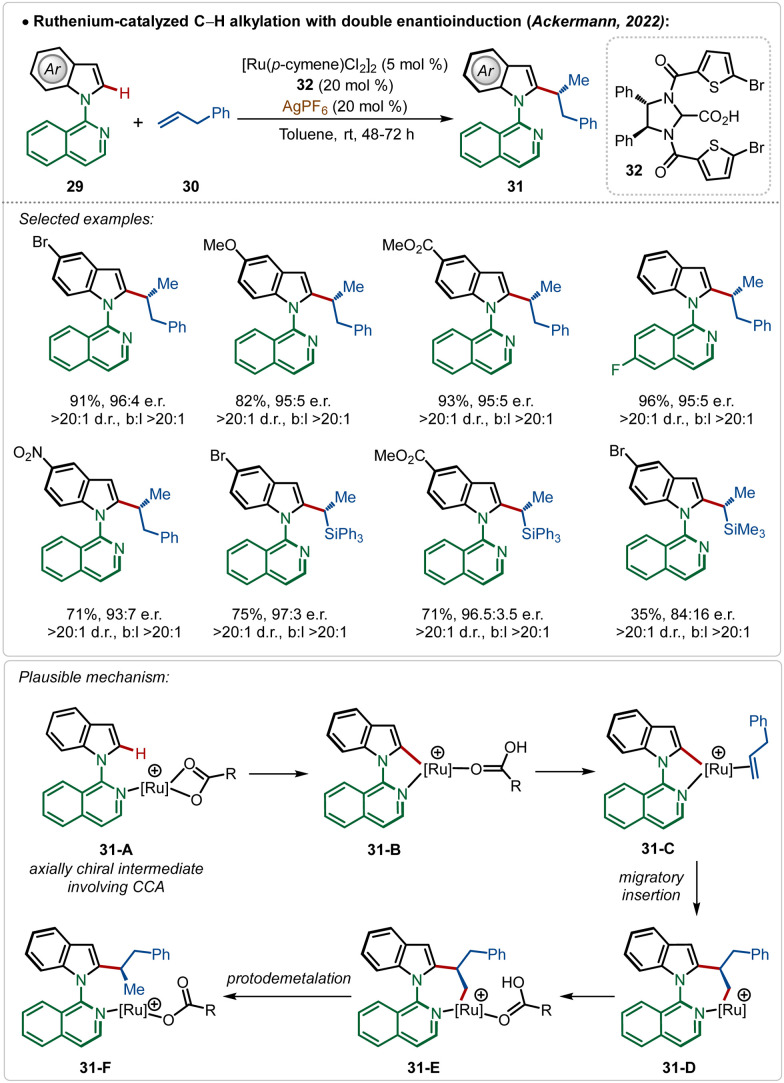
Ruthenium-catalyzed C–H alkylation with double enantio-induction.

Very recently, the Ackermann group has described an interesting example of ruthenium-catalyzed *ortho*-C–H alkylation of indole derivatives where both point and axial chirality were generated consequently. Easily accessible chiral imidazolidine carboxylic acids were used for the chiral induction (Scheme 5).^13*a*^ The strategy successfully involved diverse allyl arenes and vinyl silanes as the coupling partners in combination with different indole analogues. The chiral carboxylic acid played a decisive role in both the atroposelective C–H activation (intermediates **31-A** and **31-B**) in the C-2 position of indole and in the migratory insertion of the olefin (31-C–31-F) to generate the chiral molecules.

## 3. Rhodium catalysis with silver additives

**Scheme 6:**
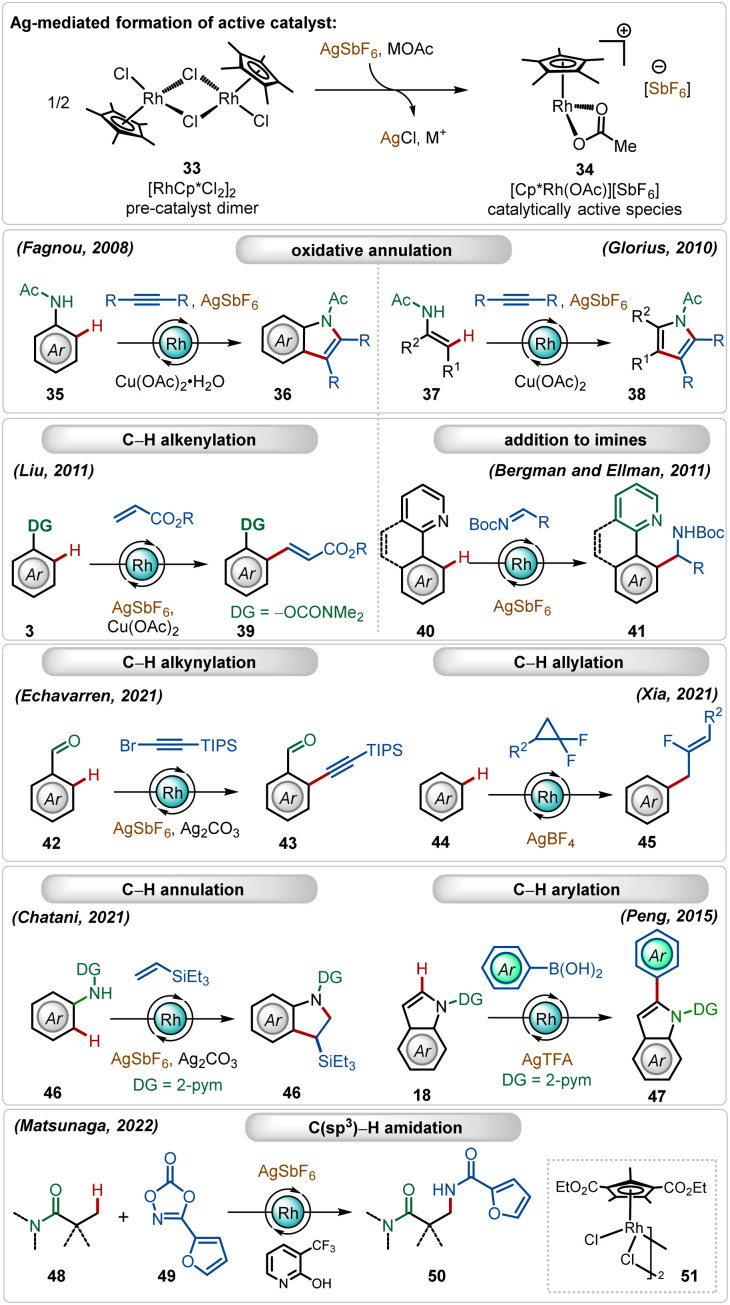
Overview of Ag-assisted [Rh]-catalyzed C–H activations.

In this expanse, rhodium-catalyzed C–H activation reactions have also exhibited tremendous potential owing to the high reactivity and versatility.^1*b*,*f*,28^ The most popular half-sandwich catalyst used in these transformations is the rhodium pentamethylcyclopentadienyl chloride dimer ([Cp*RhCl_2_]_2_). Similar to the reactivity of the ruthenium catalyst, already discussed in the preceding section, this dimer is also a pre-catalyst, which requires Ag-mediated activation to form its monomeric cationic variant.^1*b*,*f*^ In these cases, silver hexafluoroantimonate (AgSbF_6_), silver triflimide (AgNTf_2_), silver carbonate (Ag_2_CO_3_), silver tetrafluoroborate (AgBF_4_), silver acetate (AgOAc) or silver trifluoroacetate (AgTFA) are used as common additives in combination with additional carboxylate sources. These silver salts, when combined with the Rh(iii)-catalyst, mediate different C–H functionalization reactions, such as C–H alkenylation,^29^ C–H alkylation,^30^ C–H alkynylation,^31^ C–H allylation,^32^ C–H arylation,^33^*etc.* (Scheme 6).^1*b*,*f*,28^

Early examples of the efficiency of cationic Rh(iii)-complexes were demonstrated by Fagnou and co-workers through an oxidative C–H/N–H annulation of anilides with internal alkynes.^34^ Later, Glorius reported a similar Rh(iii)-catalyzed C–H/N–H annulation of enamides with internal alkynes.^35^ An important example of *in situ* generated cationic Rh(iii)-catalyzed oxidative olefination of phenols were illustrated by Liu.^36^ Ellman and Bergman showed that the combination of the [Cp*RhCl_2_]_2_ catalyst with AgSbF_6_ could also promote a Grignard like addition of C–H bonds to aldimines.^37^ Recently, Echavarren delineated alkynylation of aldehydes using alkynyl bromides as the reaction partner.^31^ In a similar trend, Xia and co-workers described the direct addition of C–H bonds to difluorocyclopropanes forming fluorinated allyl benzenes, where AgBF_4_ was utilized as a suitable additive.^32^ This Ag(i)-mediated cationic Rh(iii)-catalyzed approach was also garnered for annulation with olefins^38^ and C-2 arylation of indoles.^33^ This catalytic system is also suitable for C(sp^3^)–H bond activation reactions. Among other important findings,^39^ Matsunaga and co-workers described a C(sp^3^)–H amidation of aliphatic amides using dioxazolones as the amidating agent, where electron-deficient Rh(iii)-pre-catalyst and pyridone ligands were an imperative part of the transformation.^39*a*^

**Scheme 7:**
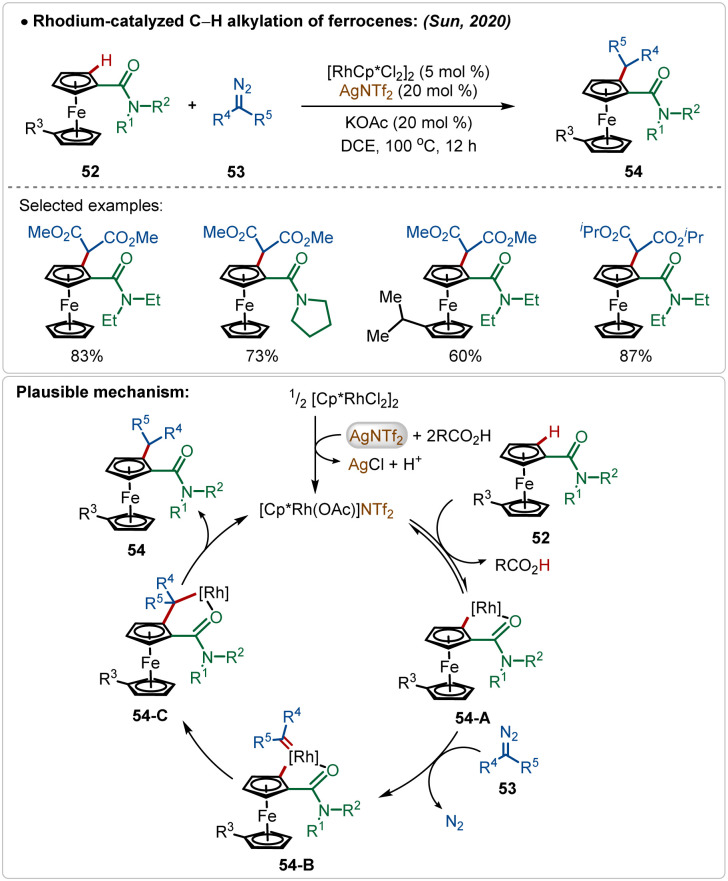
Rhodium-catalyzed C–H alkylation.

Sun and co-workers reported an elegant example of rhodium-catalyzed C–H alkylation of modified ferrocenes, where various alkylated ferrocenes were obtained in good yields (Scheme 7).^29^ In this work, AgNTf_2_ was used as the additive in combination with the KOAc co-additive. This combination successfully led to the *in situ* formation of the crucial catalytically active cationic [Rh]-species. Diazo-salts were used as the suitable alkyl source, where the reaction proceeded *via* the formation of Rh-carbenoid intermediates. An amide directed regioselective insertion of these Rh-carbenoid intermediates afforded the alkylated products.

**Scheme 8:**
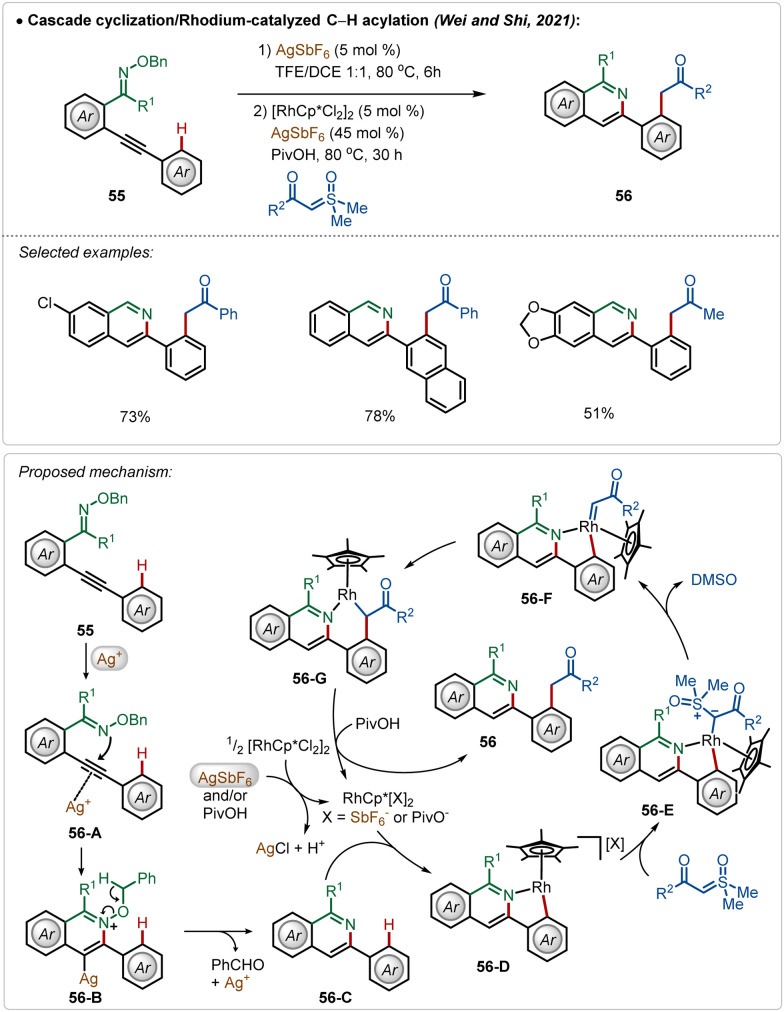
Rhodium-catalyzed C–H activation with bifocal use of AgSbF_6_.

Another interesting Rh(iii)-catalyzed C–H activation reaction was recently described by Wei, Shi, and co-workers,^40^ in which novel isoquinoline derivatives were successfully fabricated in moderate to good yields (Scheme 8). In this process, AgSbF_6_ played a dual role. First of all, the Ag(i)-salt acted as a Lewis acid to activate the internal alkyne to mediate an intramolecular cyclization with oxime ether functionality, constructing the corresponding isoquinoline analogue 56-C. Then the isoquinoline directed *ortho*-C–H bond activation of the arene took place (56-D) followed by the formation of an Rh-carbenoid species 56-F after a loss of DMSO. Next, the respective intermediate underwent a migratory insertion to generate 56-G, protodemetalation, which led to the formation of the desired product (56). In the latter case, Ag(i)-salt assisted the formation of catalytically active cationic Rh(iii)-catalyst, which enabled the C–H activation.

## 4. Iridium catalysis with silver additives

**Scheme 9:**
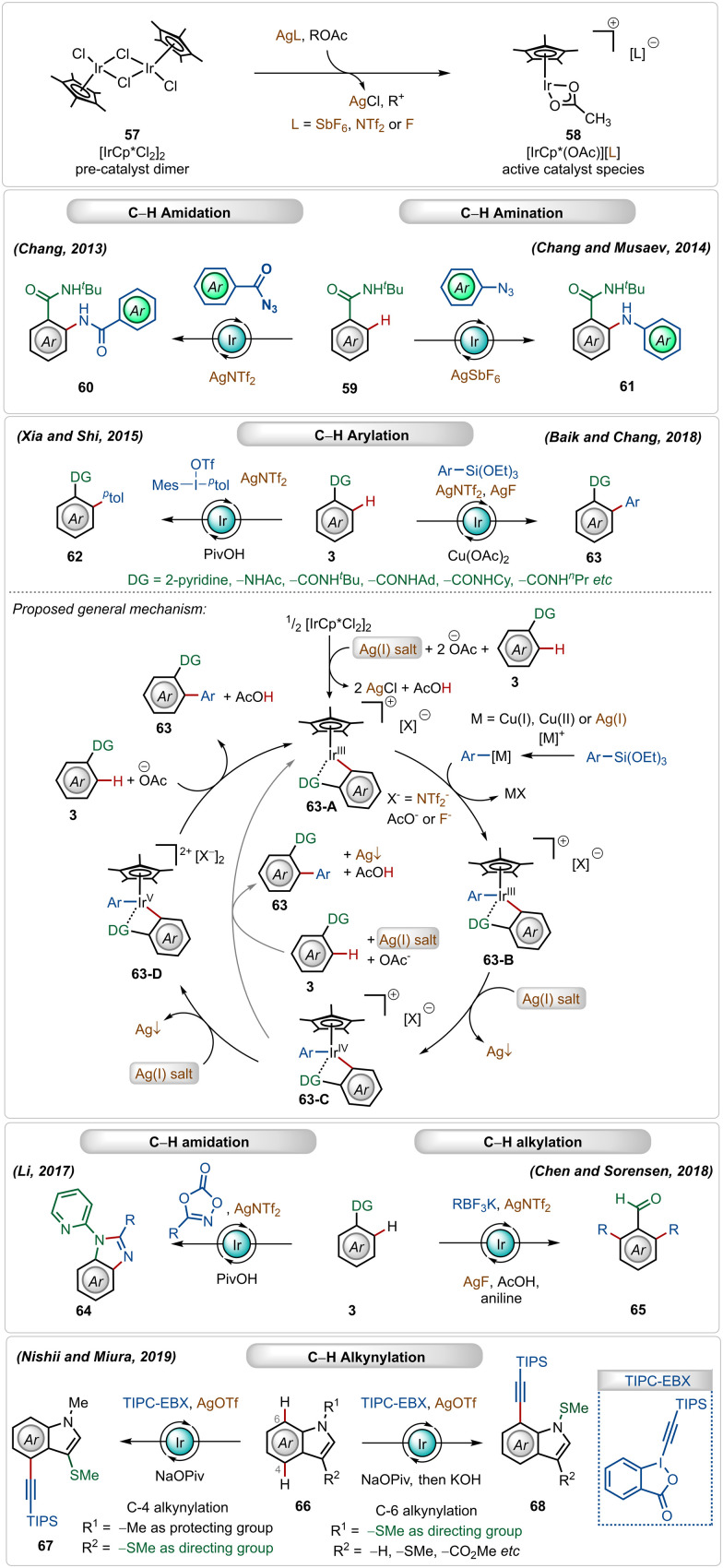
Overview of Ag-assisted [Ir]-catalyzed C–H activations.

Cationic iridium(iii)-catalysts have also found widespread application in many C–H activation reactions.^1*b*,*c*,41^ One of the main half-sandwich iridium-catalysts is the iridium pentamethylcyclopentadienyl chloride dimer ([Cp*IrCl_2_]_2_), which is similar to the above-mentioned rhodium catalyst that must be converted into its monomeric form using Ag(i)-additives.^1*b*,*c*,41^ In this case, usually used silver salt additives are silver hexafluoroantimonate (AgSbF_6_), silver carbonate (Ag_2_CO_3_), silver triflimide (AgNTf_2_), or silver fluoride (AgF).^1*c*,41^ This Ir(iii)-catalyzed strategy has been successful for a large variety of direct C–H activation reactions (Scheme 9).^41^

In 2013, Chang utilized the bench-stable [Cp*IrCl_2_]_2_ catalyst in combination with the AgNTf_2_ additive to perform *ortho*-C–H amidation of benzamides with acyl azides.^42*a*^ Later, in 2014, they also established cationic Ir(iii)-catalyzed direct C–H amination with sulfonyl azides and aryl azides as amidating agents.^42*b*,43^ The Ir(iii)-catalyst was also suitable for C–H arylation of arenes using arylsiloxanes^44*a*^ and diaryliodonium salts as the aryl source.^44*c*^ Recently, Chang and Baik also used aryl siloxanes as arylating agents for C–H arylation reactions under Ir(iii)-catalysis.^44*b*^ In both reactions, the presence of the AgNTf_2_ additive was crucial.

In 2018, Chen and Sorensen reported *ortho*-C–H alkylation of arenes utilizing alkyl trifluoroborates as the alkylating agent, where the presence of AgNTf_2_ and AgF was necessary for the optimal outcome.^45^ Nishi and Miura discovered an interesting example of an *in situ* generated cationic Ir(iii)-catalyzed regioselective alkynylation strategy, achieving C-4 and C-7 selective alkynylation of indoles.^46^

**Scheme 10:**
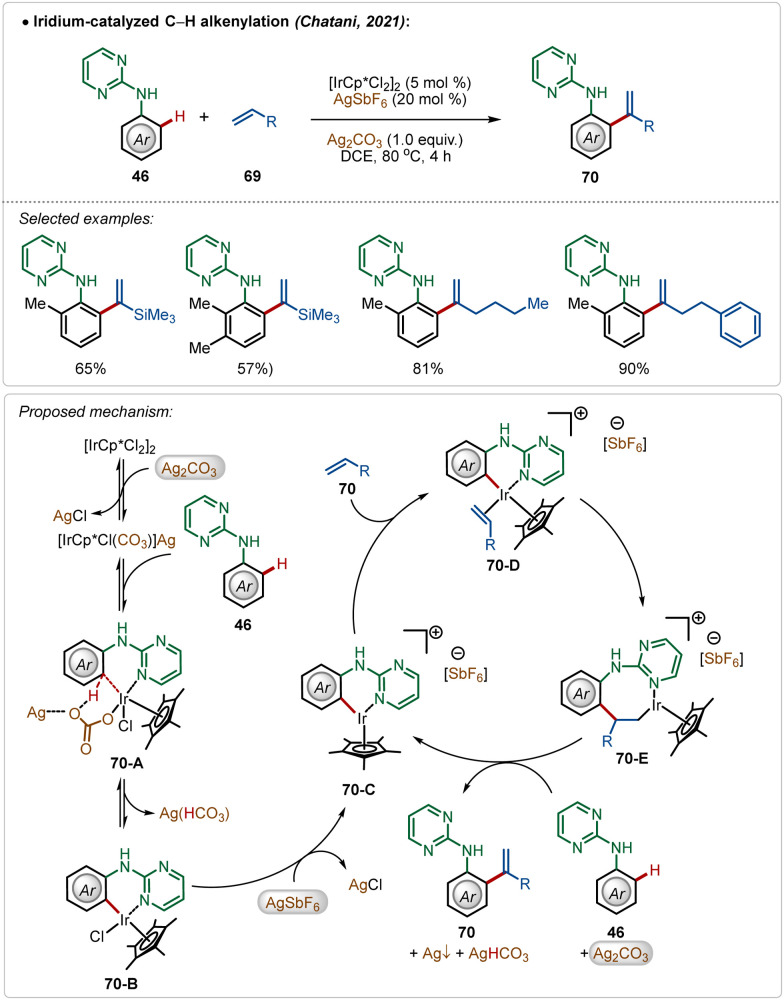
Iridium-catalyzed C–H alkenylation.

In a fascinating transformation, Chatani and co-workers disclosed an iridium-catalyzed C–H olefination strategy for the synthesis of 1,1-diaryl olefins, where both AgSbF_6_ and Ag_2_CO_3_ were used as additives (Scheme 10).^47^ Here, aniline derivatives were used as suitable substrates employing a pyrimidine-directing group, which provided access to several styrene derivatives in moderate to good yields. Unlike typical C–H olefination reactions, this process leads to branched olefins exclusively.

Based on the experimental results, DFT calculations, and literature precedents, the plausible mechanism was proposed. First, the reaction of pre-catalyst [Cp*IrCl_2_]_2_ with additive Ag_2_CO_3_ generated the intermediate [Cp*IrCl(CO_3_)](Ag). This species executed the desired C–H activation after an interaction with the substrate. The addition of AgSbF_6_ promoted the reaction by removing the other residual chloride, forming AgCl and the active species 70-C, which actively participated in the catalytic transformation. The cationic intermediate 70-C interacted with the olefin, forming intermediate 70-E after a migratory insertion, which upon β-hydride elimination gave the olefinated product (70).

**Scheme 11:**
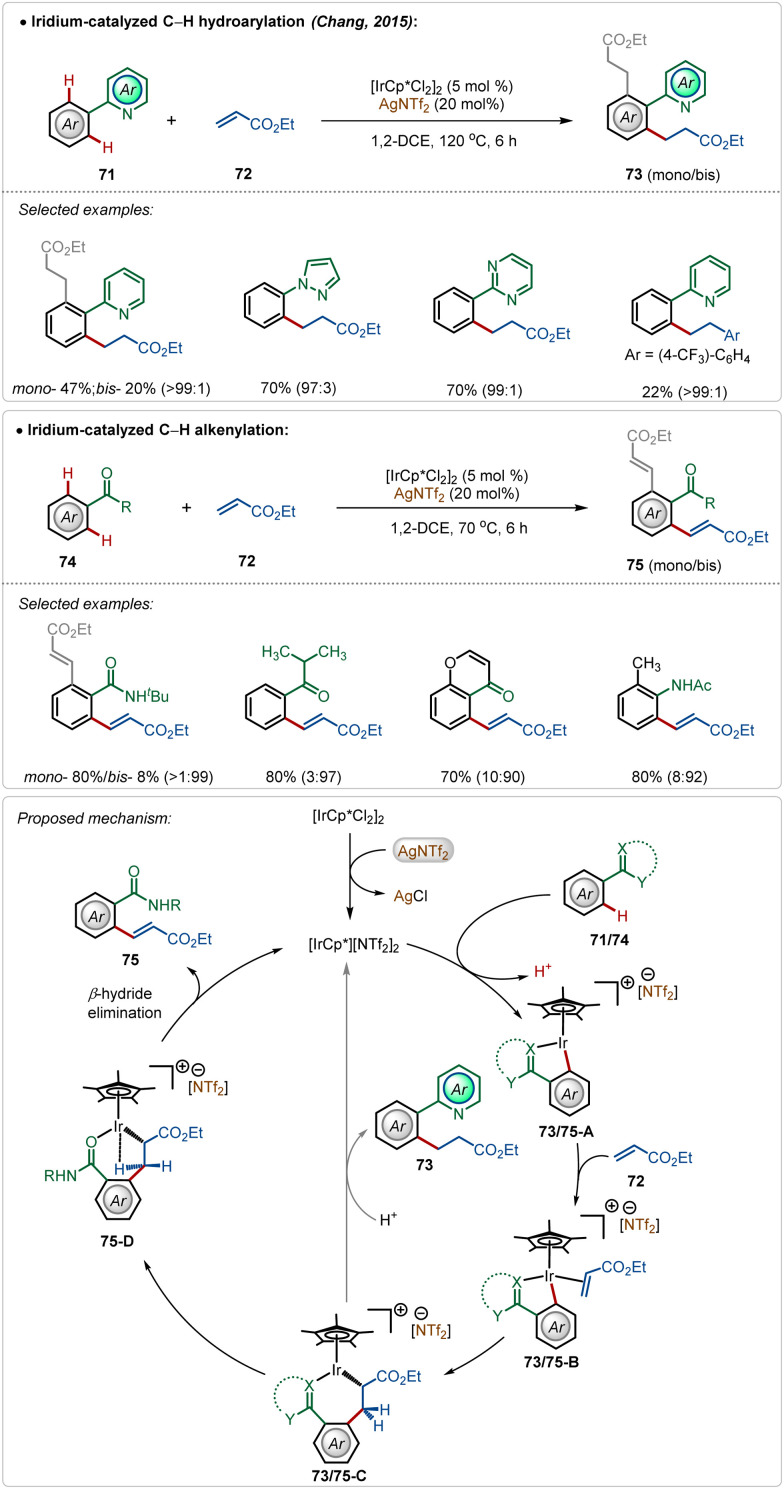
Iridium-catalyzed C–H alkenylation vs alkylation guided by directing groups.

In 2015, Chang and co-workers depicted the divergent reactivity of the Ir(iii)-catalyst through the olefination and alkylation of arenes with activated olefins, where the product selectivity was largely dependent upon the directing group (Scheme 11).^48^ Strongly coordinating pyridyl, pyrimidyl, and pyrazolyl groups led to alkylated products, whereas weakly coordinating ketone, anilide, and amide generated the olefinated products. In these transformations, AgNTf_2_ played a critical role in forming the active cationic iridium catalyst, which was responsible for the product formation.

Initial steps of the catalytic cycles, such as C–H activation, olefin coordination, and migratory insertion were the same for both *N*-centered and *O*-centered directing groups. The directing group played a decisive role in chemoselectivity. The product was formed after a structural modification of the key intermediate 73/75-C. Now the strong interaction between strongly Lewis basic *N*-centre with the Lewis acidic Ir(iii)-centre restricted the change in the orientation owing to the high rigidity of the iridacycle (73/75-C), which led to alkylation. The weakly Lewis basic *O*-centered directing group had a weaker interaction with the Ir(iii)-centre leading to a feasible change in the structural orientation, which underwent β-hydride elimination through agostic interaction to deliver the olefinated product (Scheme 11).

**Scheme 12:**
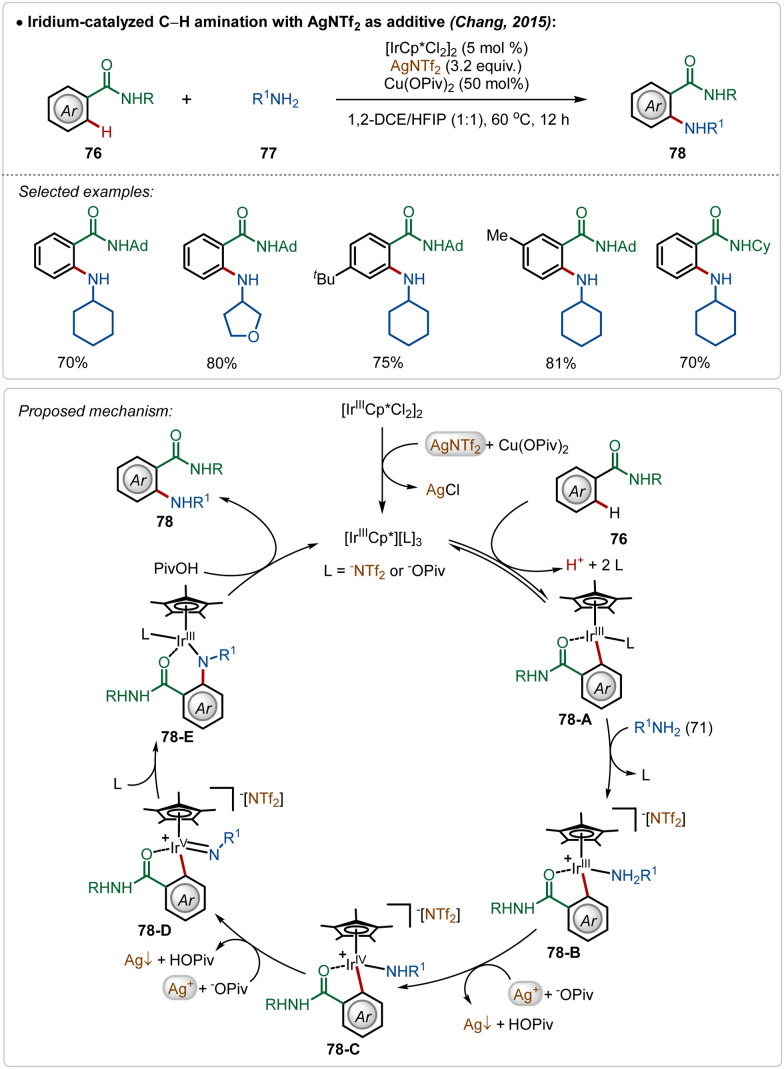
Iridium-catalyzed amination using aliphatic primary amines.

The Chang group further demonstrated an iridium catalyzed amination using alkyl amines as the amine source.^49^ Similar to earlier examples, this reaction was also promoted by a cationic Ir(iii)-catalyst, generated through the reaction between the bench-stable Ir(iii)-dimeric catalyst and AgNTf_2_. The interesting feature of this transformation was the involvement of an oxidation-induced reductive elimination process, which involved an Ir(v)-intermediate consisting of an amido ligand. The insertion on the amido-Ir(v) intermediate (78-D) followed by protodemetalation (78-E) produced the aminated product. Poor reactivity of secondary amines in this transformation further justified the involvement of an oxidation-induced reductive elimination pathway instead of a direct reductive elimination from the Ir(iii)-intermediate (Scheme 12).

**Scheme 13:**
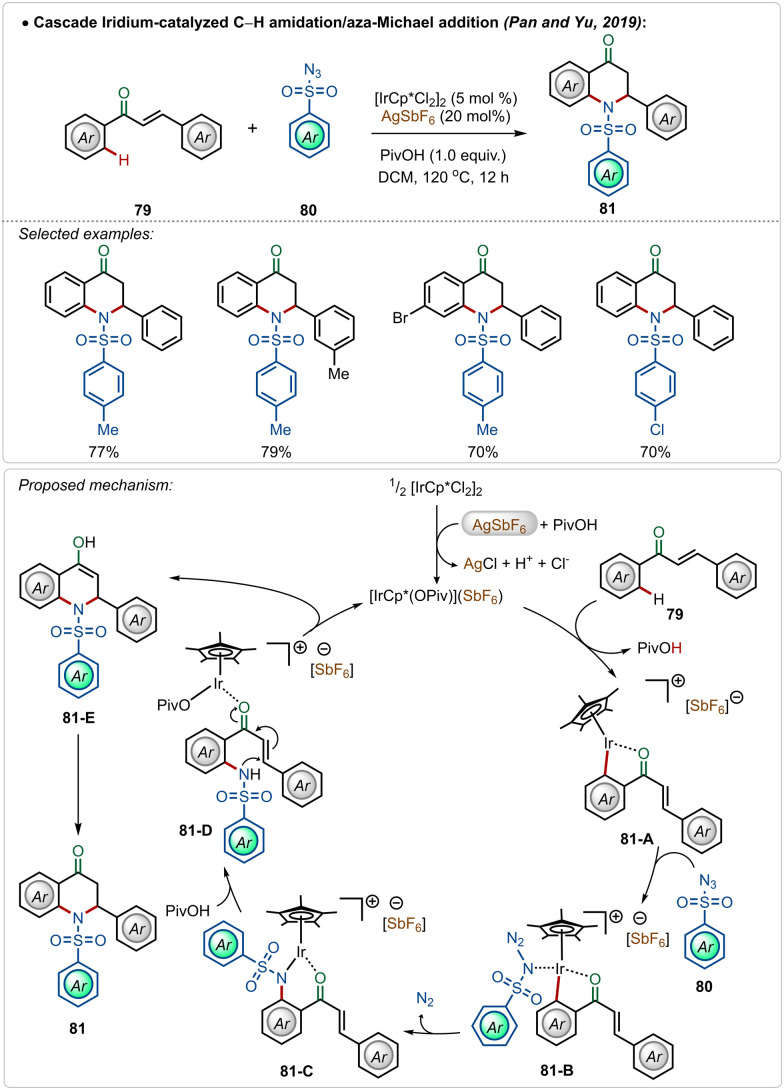
Iridium-catalyzed C–H amidation/aza-Michael addition cascade.

A unique dihydroquinolinone synthesis was depicted by Pan, Yu and co-workers through an iridium-catalyzed C–H amidation followed by an intramolecular aza-Michael addition strategy.^50^ Starting from a large variety of chalcones, through this one-pot procedure different cyclic dihydroquinolinone derivatives were accessed in good yields, where two new C–N bonds were successfully formed (Scheme 13). The proposed mechanism played a similar role to AgSbF_6_, which in combination with pivalic acid, led to the formation of the active iridium-species. After C–H activation followed by the nitrene insertion, the amidated product 81-D was formed, which underwent a facile Michael addition to the α,β-unsaturated ketone moiety to form the heterocyclic dihydroquinolinone product.

## 5. Palladium catalysis with silver additives

In the C–H activation regime, palladium-catalysis remains at the forefront owing to its diverse applicability in proximal and distal C(sp^2^)–H and C(sp^3^)–H bond functionalization.^51^ Notably, many of these transformations require Ag-additives to obtain the optimal outcome.^5*b*–*d*^ Typically, these additives play a crucial role as a terminal oxidant to enable the catalytic turnover.^51^

**Scheme 14:**
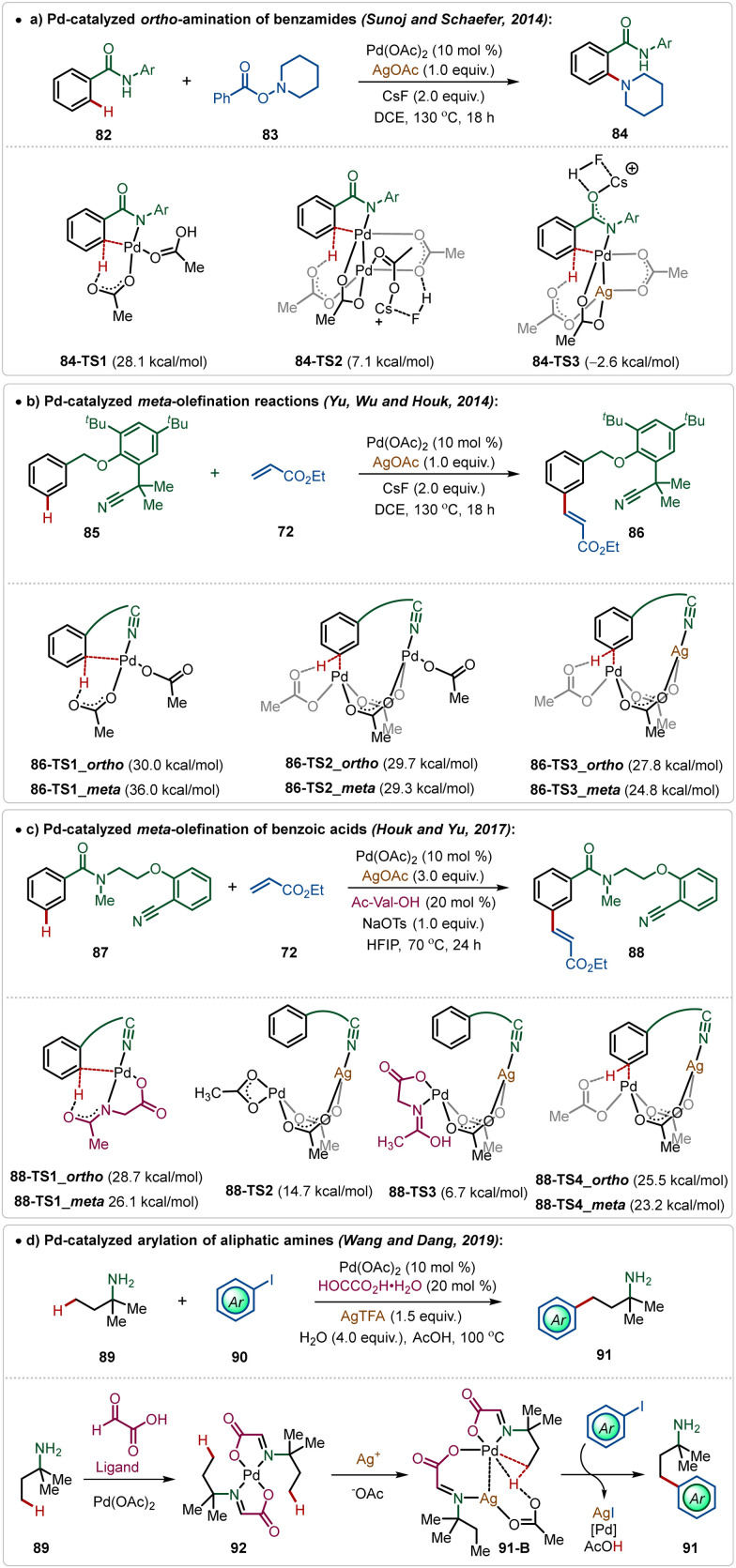
Overview of Ag-assisted [Pd]-catalyzed C–H activations.

However, recently, Larrosa has depicted that phosphine ligated Ag(i)-carboxylates could also promote C–H activation of electron-deficient arenes forming Ag-aryl intermediates, which then realized transmetalation with a Pd-intermediate to accomplish the arylation with aryl iodides.^52^ Recent findings have further reported some interesting insights into Pd-catalyzed C–H activation reactions promoted by Ag-additives, which divulges the critical role of a Pd–Ag heterobimetallic intermediate species in these transformations.^5*b*–*d*^ For example, Pd(ii)-catalyzed *ortho*-amination reaction of benzamides, which follows a Pd(ii)/Pd(iv) pathway, does not necessarily need an additional Ag-oxidant for product formation.^9*a*^ However, experimental findings suggested an indispensable role of AgOAc in this reaction. Theoretical studies by Sunoj and Schafer unveiled the formation of an energetically favourable Pd–Ag heterometallic intermediate during the process, which necessitated the use of Ag(i)-salt in this transformation (Scheme 14a).^9^

Similarly, Houk and Yu disclosed that the Pd-catalyzed distal C(sp^2^)–H olefination reaction was enabled by the formation of critical Pd–Ag heterometallic intermediates.^53^ Based on their findings, it was apparent that the Pd-monomeric complex generated exclusive *ortho*-selectivity, while the Pd-dimeric complex led to a ∼3 : 1 *meta*/*ortho* regio-isomeric mixture. Interestingly, the incorporation of an isoelectronic Ag(i)-species instead of Pd(ii)-species stabilized the transition states and elevated the *meta*-selectivity. The direct coordination of Ag(i)-species with the nitrile functionality of the directing group was responsible for the superior *meta*-selectivity (Scheme 14b).

This conceptual basis was also applicable for the template-assisted *meta*-olefination of benzoic acid derivatives.^54^ Though the transformation was ineffective in the absence of *mono*-protected amino acid (MPAA) ligands, computational studies supported the formation of a Pd–Ag bimetallic transition state and MPAA was found to stabilize this species (Scheme 14c).

The formation of Pd–Ag bimetallic clusters was also found in Pd-catalyzed C(sp^3^)–H bond activation reactions. Dang and Wang showed a Pd-catalyzed C(sp^3^)–H arylation of aliphatic amines, which was enabled by the formation of Pd–Ag heterodimeric species consisting of carboxylate bridges (Scheme 14d).^55^

**Scheme 15:**
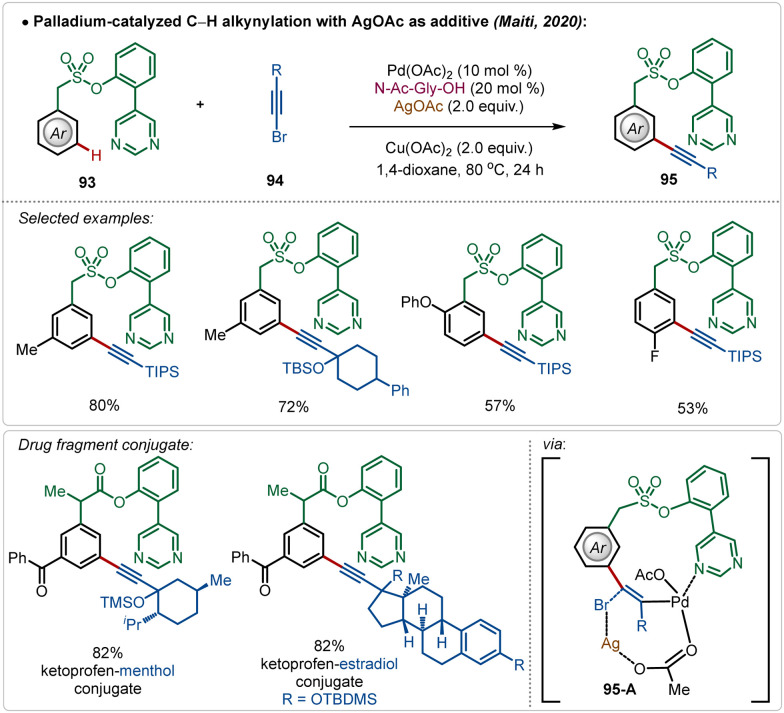
Ag-assisted [Pd]-catalyzed C–H alkynylation.

Maiti also reported the *meta*-alkynylation of arenes, where the formation of a Pd–Ag bimetallic cluster was the key for product formation.^56^ Noticeably, in this case the directing group was directly coordinated to the Pd(ii)-species, which was connected to the Ag(i)-centre through an acetate bridge. The halophilic Ag(i)-species assisted bromide removal from the alkynyl bromide in the transition-state (95-A) and thus influenced product formation (Scheme 15).

## 6. Cobalt catalysis with silver additives

In recent years, there is a surge in interest towards the development of more practical ways to perform C–H activation reactions reducing the undesired barrier between the academic and industrial research.^1^ This can be achieved by replacing expensive ruthenium-, rhodium-, iridium- and palladium-catalysts with cheap, comparatively less toxic, and Earth-abundant 3d transition metal catalysts.^1*e*^

**Scheme 16:**
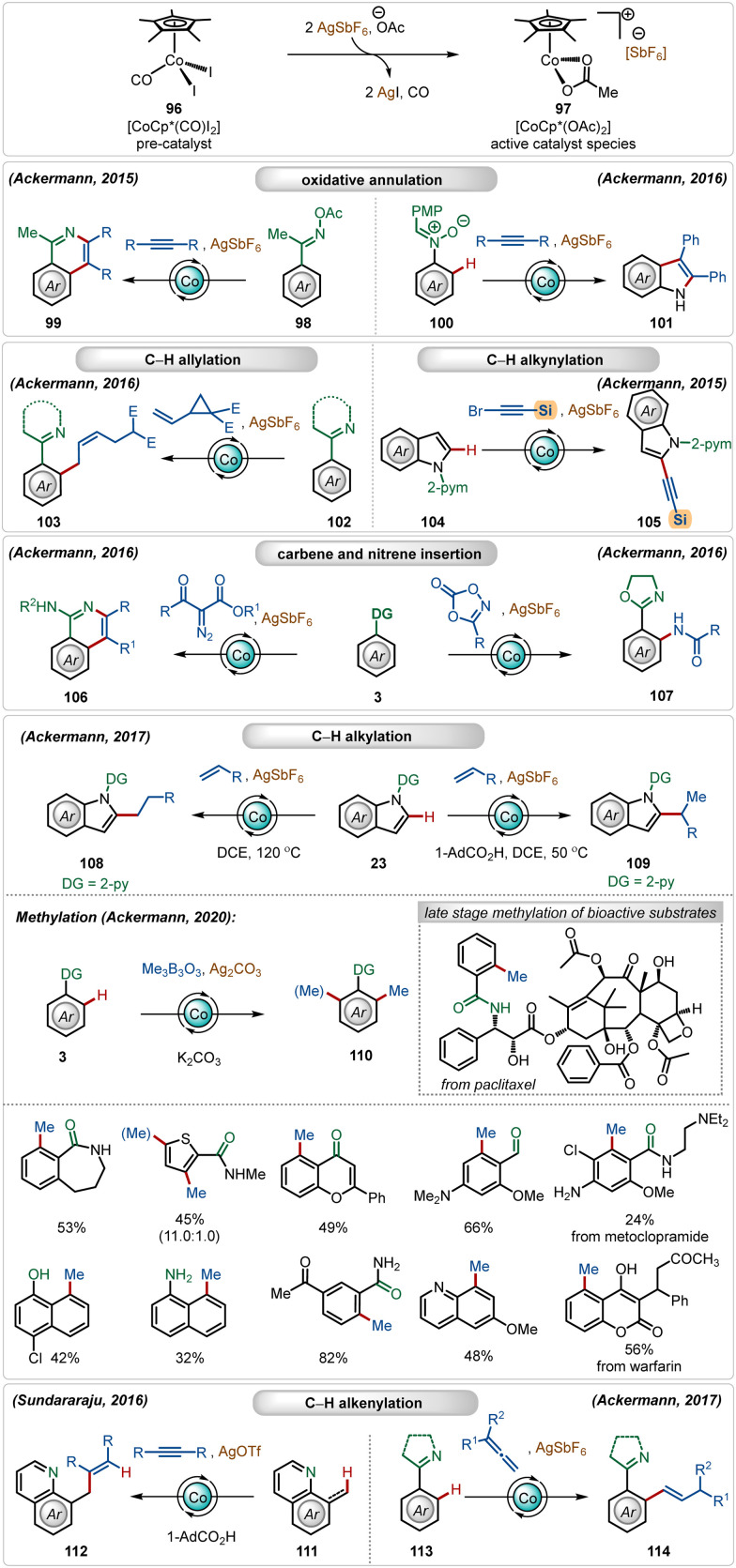
Overview of Ag-assisted [Co]-catalyzed C–H activations.

In this domain, among all the possible 3d transition metals, currently the most used transition-metal catalyst is cobalt.^57,58^ The most common cobalt catalyst used in C–H activation reactions is the half-sandwich complex [Cp*Co(CO)I_2_].^57,58^ Although it is already a monomeric catalyst, it is less reactive and mainly acts a pre-catalyst. Therefore, it usually requires the presence of Ag-based additives to exchange its iodide ligands for the formation of reactive cationic species.^1*b*^ In this case, silver carbonate, silver hexafluoroantimonate (AgSbF_6_), silver acetate (AgOAc), or silver triflate (AgOTf) are common additives. This combination under the optimal reaction conditions has been applied to a diversity of transformation, such as C–H alkylation,^59^ C–H carboamination,^60^ C–H alkenylation,^61^ C–H alkynylation,^62^ C–H allylation,^63^ and C–H annulation^64^ (Scheme 16). A notable contribution has been documented by the Ackermann group,^65^ where they described a versatile Co(iii)-catalyzed oxidative annulation with internal alkynes with *O*-acetyl oximes^65*l*^ and nitrones^65*i*^ without using external oxidants. They also demonstrated an effective application of the *in situ* generated cationic Co(iii)-catalyst for a selective synthesis of *Z*-olefins, where vinyl cyclopropanes were used as the reaction partners.^65*g*^ This catalytic manifold was also suitable for C-2 alkynylation of indoles with alkynyl bromides. Among other notable findings, Ackermann and co-workers also established the C–H/N–H annulation reaction involving *α*-diazocarbonyl compounds as the carbene precursor^65*h*^ and C–H amidation with dioxazolones as the nitrene source.^65*f*^ They also discovered a versatile C-2 alkylation of indoles with full selectivity control.^59*e*^ The selectivity was governed by the carboxylic acid additive. In the presence of acid, branched alkylation was observed under mild conditions, whereas in the absence of the carboxylic acid linear alkylation was the exclusive reaction. In both cases, the AgSbF_6_ additive played a critical role in generating the catalytically active Co(iii)-catalyst. Later, the Ackermann group also demonstrated a versatile *ortho*-methylation strategy using a Co(iii)-sandwich complex [Cp*Co(C_6_H_6_)](PF_6_)_2_.^59*a*^ This Co(iii)-catalyzed methylation strategy enabled the methylation of a large variety of arene analogues, tolerating different weakly as well as strongly coordinating directing groups. Furthermore, the catalytic conditions smoothly executed late-stage methylation of several structurally complex natural products and drug molecules. Sundararaju and co-workers reported an early example of Co(iii)-catalyzed C(sp^3^)–H olefination through hydroarylation of alkynes.^61^ Another attractive allene hydroarylation strategy was unveiled by Ackermann, offering modular access to alkenylated arenes.^65*e*^

**Scheme 17:**
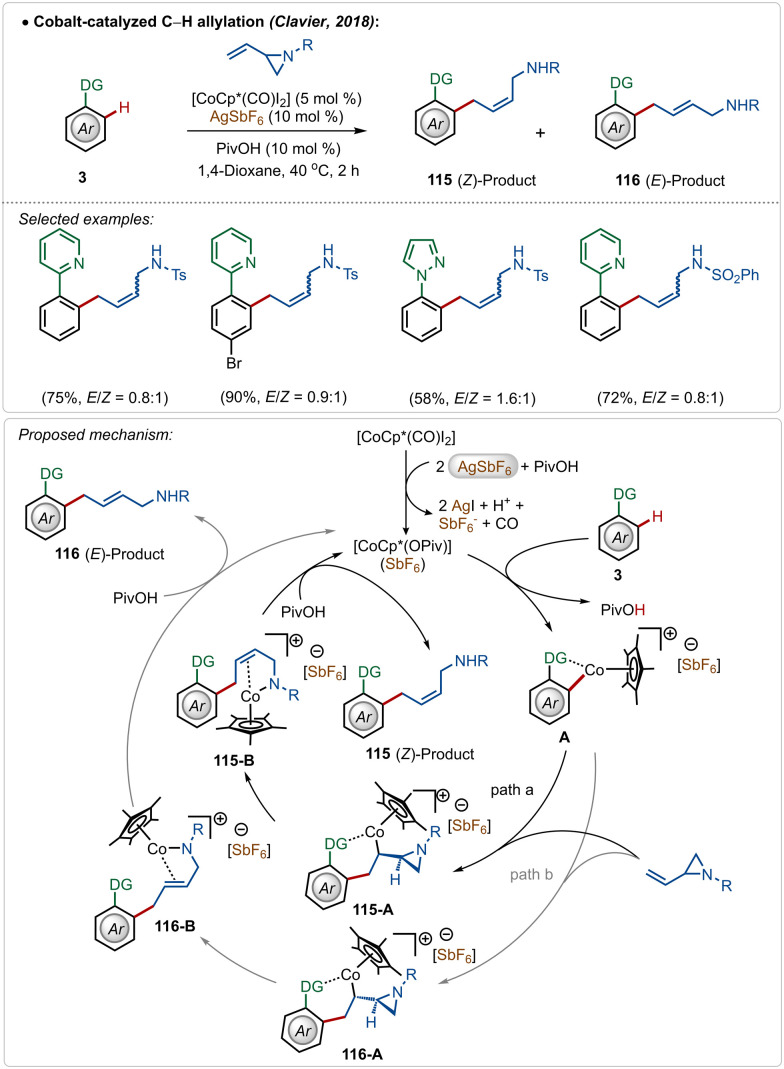
Cobalt-catalyzed C–H allylation with vinylaziridines.

Clavier and co-workers described a cobalt-catalyzed C–H allylation of arenes with vinyl aziridines (Scheme 17).^66^ A separable mixture of (*E*/*Z*)-alkenes was achieved in a proportion close to 1 : 1 in most cases.

Although the method presented a low selectivity, this strategy worked efficiently on different substrates with strongly coordinating 2-pyridyl and 1-pyrazolyl directing groups. It was initiated with the known modification of the pre-catalyst [Cp*Co(CO)I_2_] to its active cationic form [Cp*Co(OPiv)](SbF_6_) with the aid of AgSbF_6_. Then the active species interacted with the substrate to form metallacycle intermediate A through C–H activation. It then realized olefin-insertion on both possible faces of the coupling partner, and this difference led the mechanism to slip into path “a” and “b”. Usually there was no preferential pathway, which led to the formation of both the isomers in a low selectivity. A subsequential β-*N*-elimination and ligand exchange governed the final (*Z*)- and (*E*)-products respectively and regenerated the active cobalt-catalyst.

**Scheme 18:**
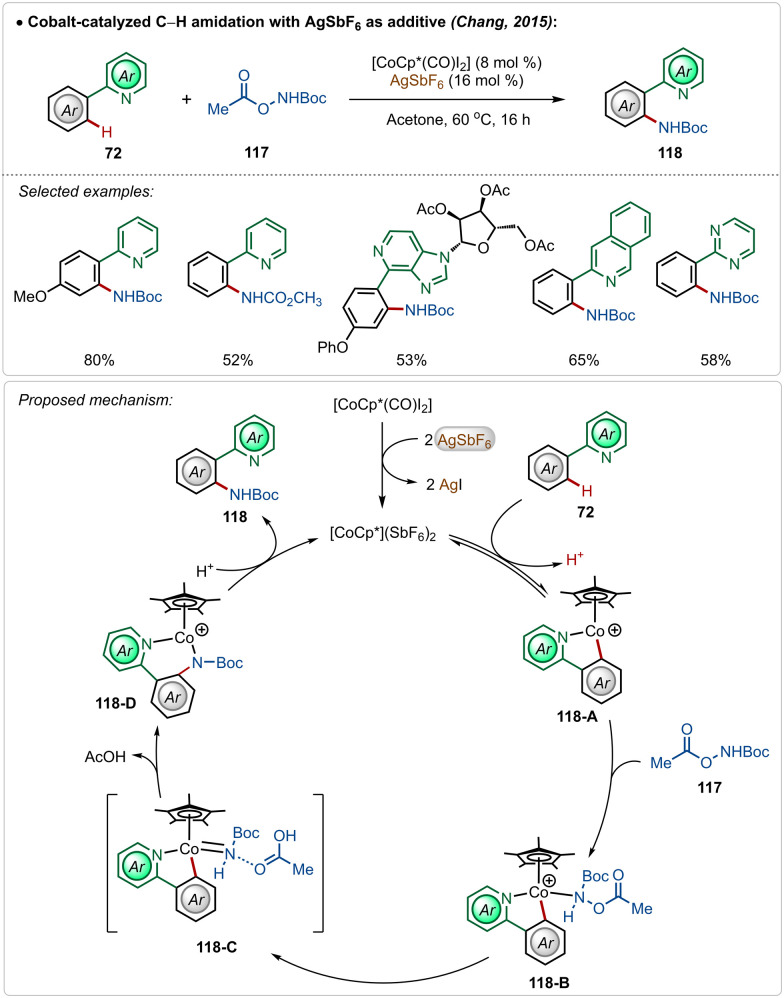
Cobalt-catalyzed C–H amidation.

In 2015, Chang and co-workers delineated Co(iii)-catalyzed amidation of 2-arylpyridines (Scheme 18).^67^ The reaction was enabled by a Co(iii)-cationic complex, formed *in situ* with the aid of AgSbF_6_. The active cationic complex on the exposure of 2-arylpyridines generated the cyclometalated intermediate 118-A, which then delivered the desired product by reacting with the amidating agent through intermediates 118-B–118-D. The amidation step plausibly involved a Co(iii)-amido intermediate, while the possible involvement of a Co(v)-nitrenoid intermediate in the transformation could not be overlooked.

**Scheme 19:**
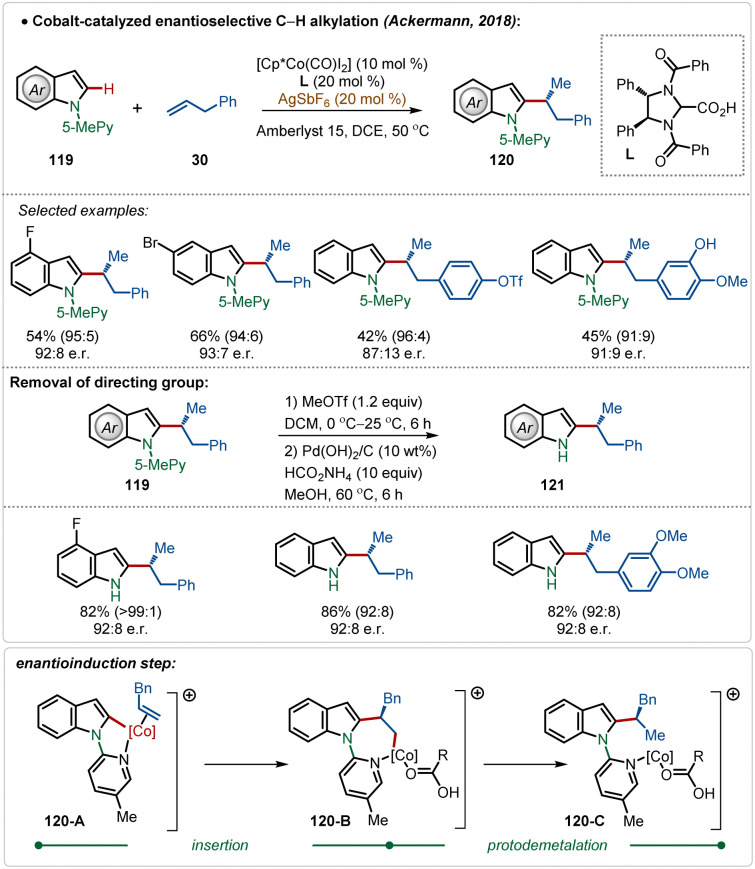
Cobalt-catalyzed enantioselective C–H alkylation.

In 2018, Ackermann and co-workers described the first example of high-valent cobalt-catalyzed enantioselective *ortho*-C–H alkylation of indoles (Scheme 19).^59*d*^ The enantioselectivity was realized using easily accessible imidazolidine carboxylic acid analogue, which was induced during the insertion and protodemetalation through the direct coordination of the chiral carboxylic acid (104-B and 104-C). Even in this transformation the role of AgSbF_6_ was critical to form the catalytically active species. This enantioselective alkylation strategy involved diverse indole and allyl arene analogues to construct the corresponding alkylated products in good yields and selectivity. The pyridyl directing group was also removed without affecting the enantiomeric excess after the Co-catalyzed transformation, obtaining free indoles in high yields.

## 7. Ag-additive free C–H activation strategies

Modern organic synthesis focuses on more practical and sustainable means to execute synthetic transformations. Avoiding the necessary additives from these transformations can be extremely effective, as it makes the approach more atom economical and reduces the trace-metal contamination on the final product.^68^ In this context, Ackermann and co-workers have disclosed several strategies using Ru,^69^ Rh,^70^ and Ir^71^ catalysts, where the need for otherwise indispensable Ag-additives can be obviated by harvesting renewable electricity.^68*c*–*f*^

**Scheme 20:**
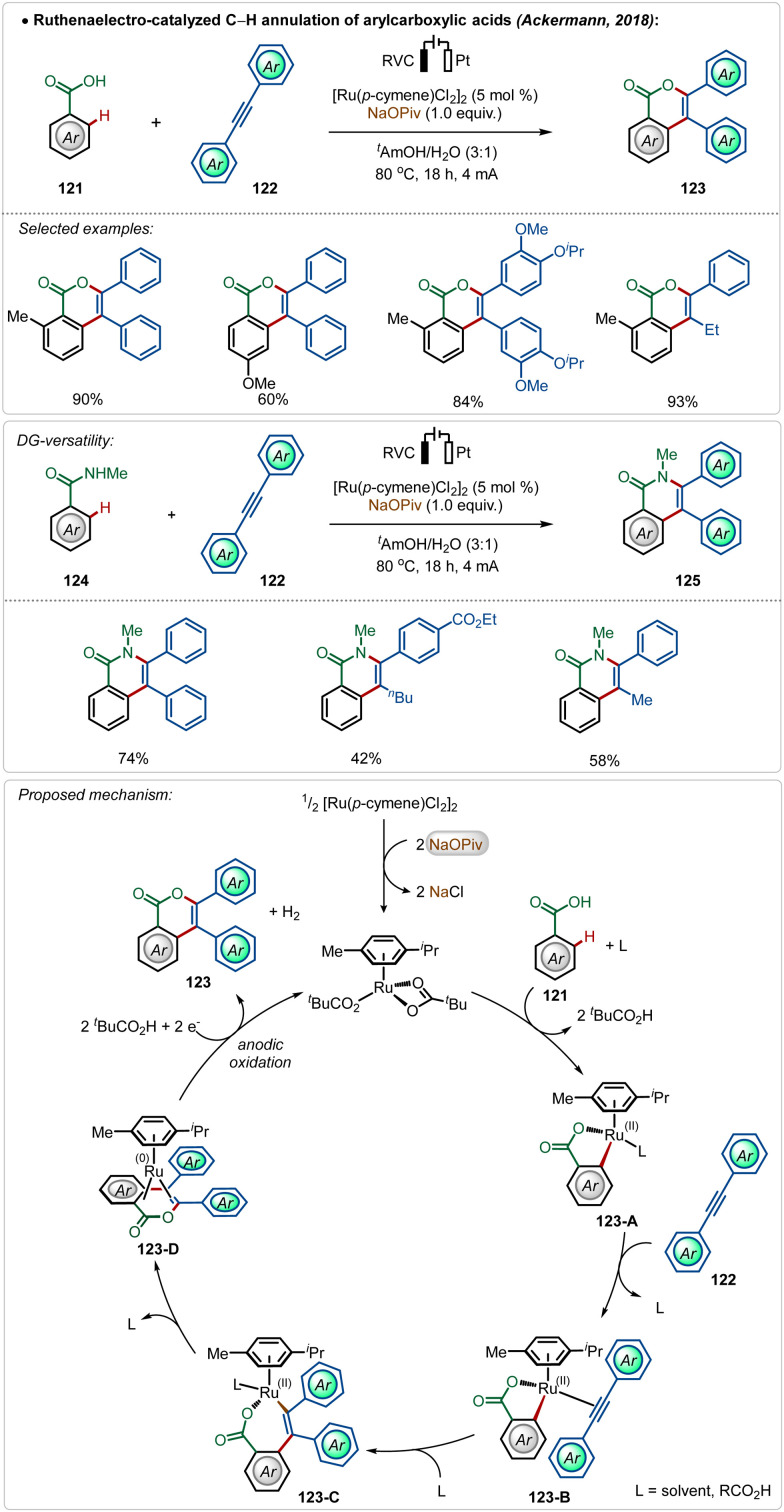
Electrooxidative ruthenium-catalyzed annulation.

In their pioneering findings, Ackermann described that the need for the AgSbF_6_ salt could be avoided when the C–H activation was performed electrochemically.^69*c*^ The reaction did not involve a cationic Ru(ii)-catalyst and was enabled in an aqueous medium. Anodic oxidation of Ru(0)-intermediate 123-D regenerated the active Ru(ii)-catalyst and the cathodic reduction of protons completed the electrochemical process with the generation of hydrogen gas. This electrochemical approach was possible with both arene carboxylic acids as well as amides and possessed high functional group tolerance leading to a broad scope (Scheme 20).

**Scheme 21:**
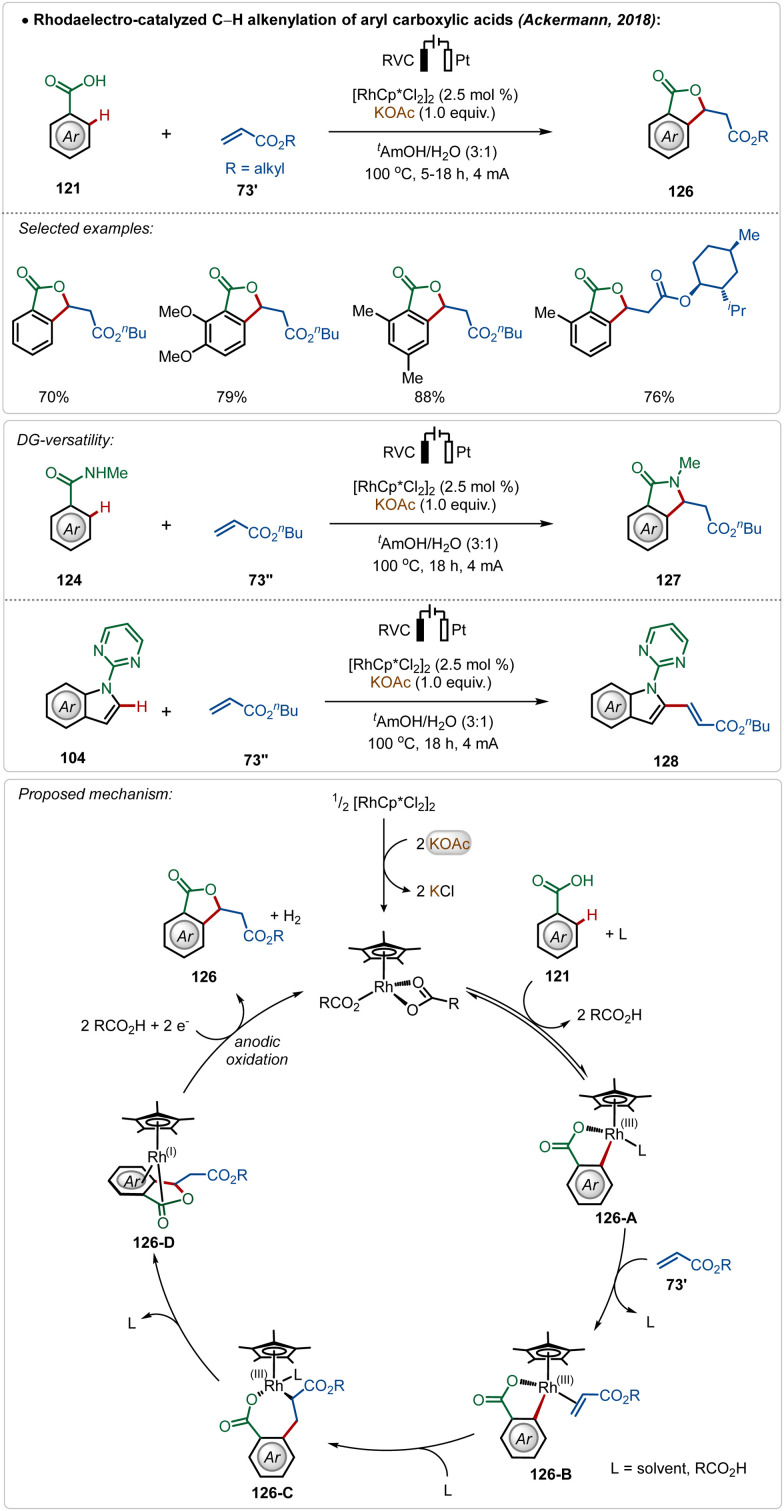
Rhodaelectro-catalyzed Ag-free C–H alkenylation of arylcarboxylic acids.

Ackermann and co-workers further reported the first Rh(iii)-catalyzed oxidative olefination/annulation strategy with weakly coordinating carboxylic acids and benzamides.^70*c*^ In contrast to the prior Ru(ii)-catalyzed processes, this transformation underwent smoothly without otherwise necessary Ag(i)-salt additives. The reaction was achievable in an aqueous medium and coupled with the hydrogen evolution reaction as the cathodic half-reaction. The strategy was versatile and accommodated a diverse range of arenes in this transformation. Even, C-2 olefination of indole was also demonstrated utilizing similar electrochemical reaction conditions. The key step enabled by electricity was the anodic oxidation of the Rh(i)-intermediate (126-D), formed *in situ* after β-hydride elimination (Scheme 21).

**Scheme 22:**
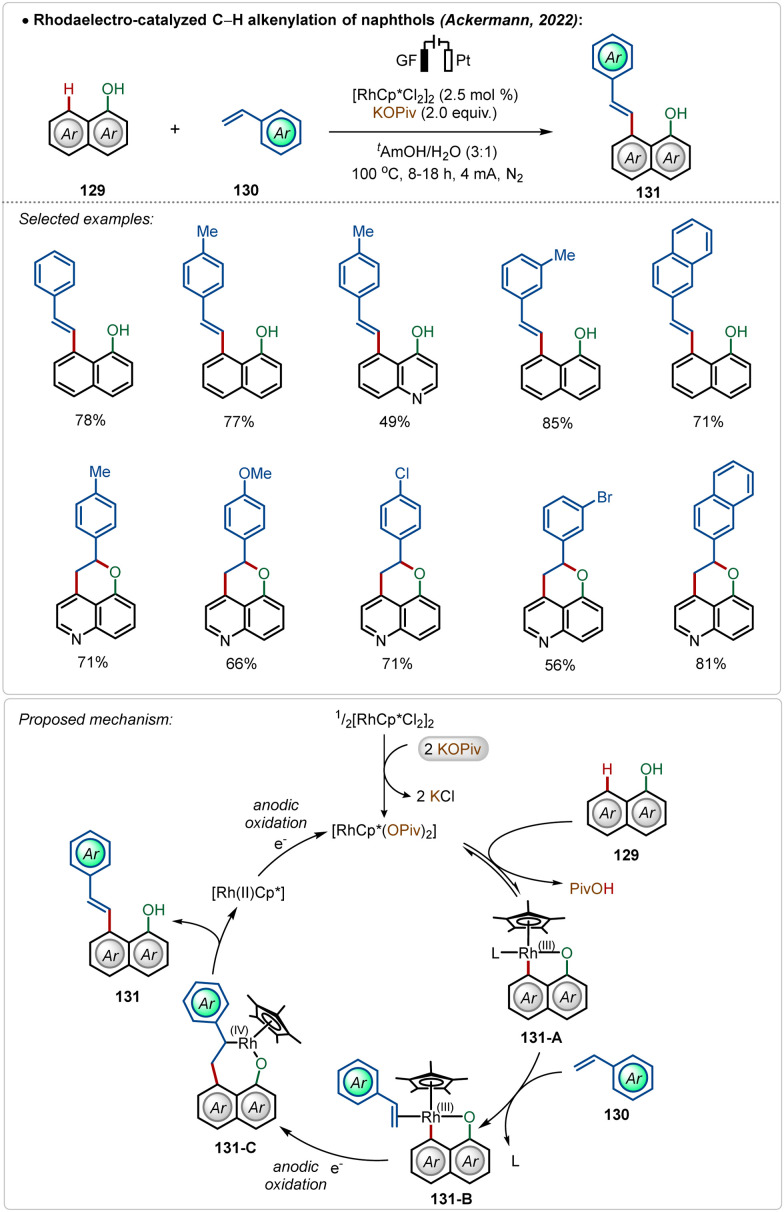
Rhodaelectro-catalyzed Ag-free C–H alkenylation of naphthols.

Later, they also detailed an elegant rhodaelectro-catalyzed C–H alkenylation at the C-8 position of naphthol with styrene-derivatives.^70*b*^ This methodology offered direct access to a broad range of C-8 olefinated naphthols in good to excellent yields. Interestingly, 5-hydroxyquinoline under electrochemical conditions furnished annulated products in moderate to good yields, displaying the versatility of the approach. Based on DFT calculations a mechanism was proposed starting with the activation of the pre-catalyst, [Cp*RhCl_2_]_2_, mediated by the additive potassium pivalate. The coordination of the hydroxy group guided the C–H activation at the C-8 position of the substrate, resulting in intermediate 131-A. A sequence of coordination of the coupling partner and migratory insertion led to the respective cyclometalated intermediate, which suffered an oxidatively induced β-hydride elimination from Rh(iv)-intermediate 131-C, leading to the product 131 and a Rh(ii) species. Anodic oxidation regenerated the active Rh(iii)-catalyst (Scheme 22). In previous examples, such reactions were performed in the presence of silver(i) salts along with an additional oxidant.^72^ Here renewable electricity was used as the terminal oxidant in the absence of Ag-salt additives, which makes these processes more general, sustainable, and resource-economic.

**Scheme 23:**
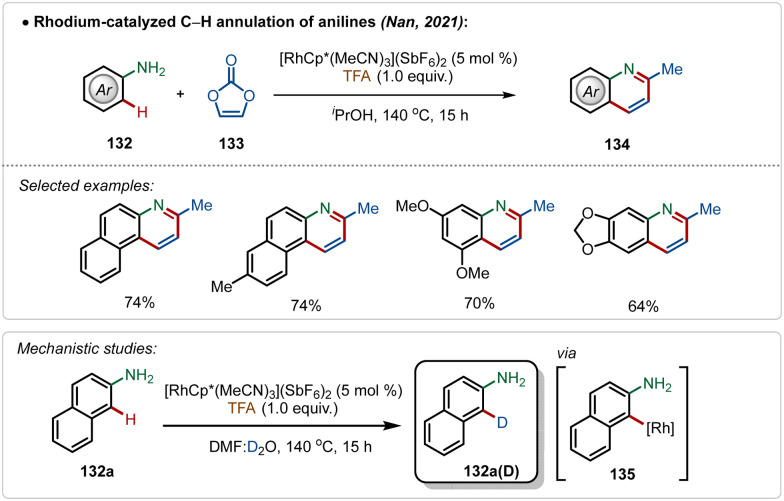
Rhodium-catalyzed Ag-free C–H annulation of anilines.

Another convenient approach for silver-additive free C–H activation is the use of a preactivated electrophilic catalyst consisting of ligands that are labile enough to facilitate the interaction between the substrate and the metal centre,^73^ which was well exemplified by the work by Nan and co-workers.^73*a*^ They delineated a successful rhodium-catalyzed C–H annulation of anilines for the formation of quinoline derivatives in moderate to good yields (Scheme 23). This strategy was empowered by the cationic [Cp*Rh(MeCN)_3_](SbF_6_)_2_ catalyst. The plausible mechanism involved an aryl-rhodium intermediate 135, probably achieved through a direct electrophilic metalation with the substrate.

**Scheme 24:**
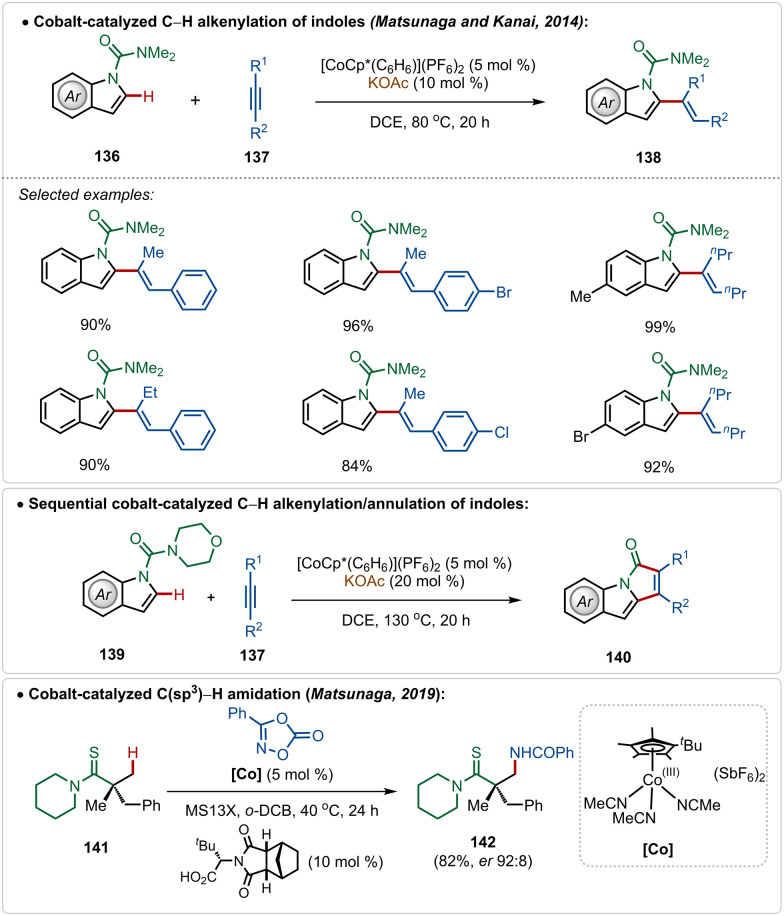
Cobalt-catalyzed Ag-free C–H alkenylation/annulation of indoles.

In fact, Co(iii)-catalyzed C–H activation is also viable in the absence of Ag(i)-additives.^73*g*–*k*^ In 2014, Matsunaga and Kanai depicted an interesting cobalt-catalyzed C–H alkenylation of indoles with alkynes, constructing C-2 olefinated indoles in good yields (Scheme 24).^74^ This olefination reaction avoided the use of an additional Ag(i)-additive and used the preactivated cationic [Cp*Co(C_6_H_6_)](PF_6_)_2_ catalyst, which in the presence of KOAc smoothly executed the transformation. Plausibly, an active cationic [Cp*Co(OAc)]^+^ species was responsible for the transformation. A sequential annulation was also realized with morpholine derived amide directing group, where the catalytic conditions successfully engendered pyrroloindolone derivatives in good yields (Scheme 24).

**Scheme 25:**
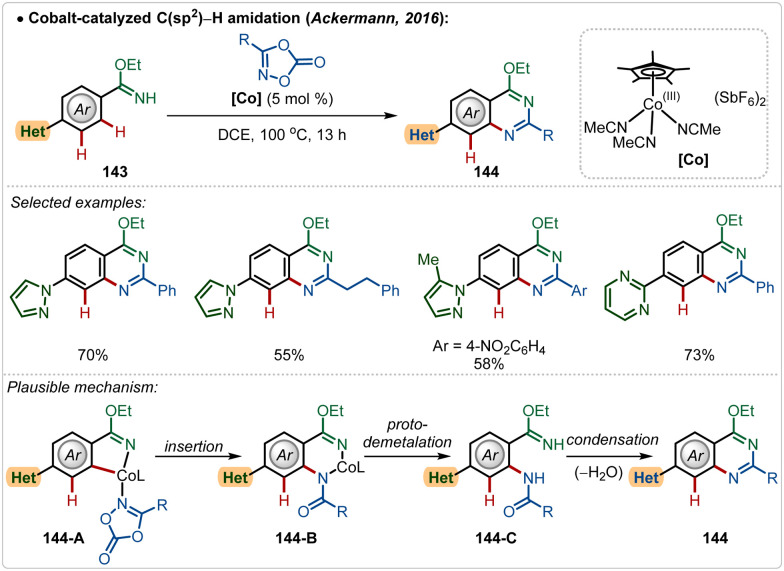
Overcoming the limitation of strongly coordinating *N*-heterocyles through Co(iii)-catalysis.

Later, in 2019, they also revealed that this silver free approach was also feasible to perform enantioselective C(sp^3^)–H amidation reaction, where the chiral carboxylic acid was involved for chiral induction.^75^ With their continuous effort on 3d transition-metal catalysis, the Ackermann group has also contributed in Cp*Co(iii)-catalyzed reactions under Ag(i)-additive free conditions. In 2016, they established a Co(iii)-catalyzed *ortho*-C–H amidation of arenes, where various *N*-heterocycle susbtitutions were tolerated under the catalytic conditions and the functionalization was enabled selectively by the imidate directing group (Scheme 25).^76^ Different quinazoline derivatives were easily accessed in good to excellent yields through this approach. Easily accessible dioxazolones were used as the amidating agents, which after insertion to the cobaltacycle intermediate 144-A delivered the *ortho*-amidated intermediate. Intermediate 144-C after intramolecular condensation with the imidate formed the quinazoline products.

**Scheme 26:**
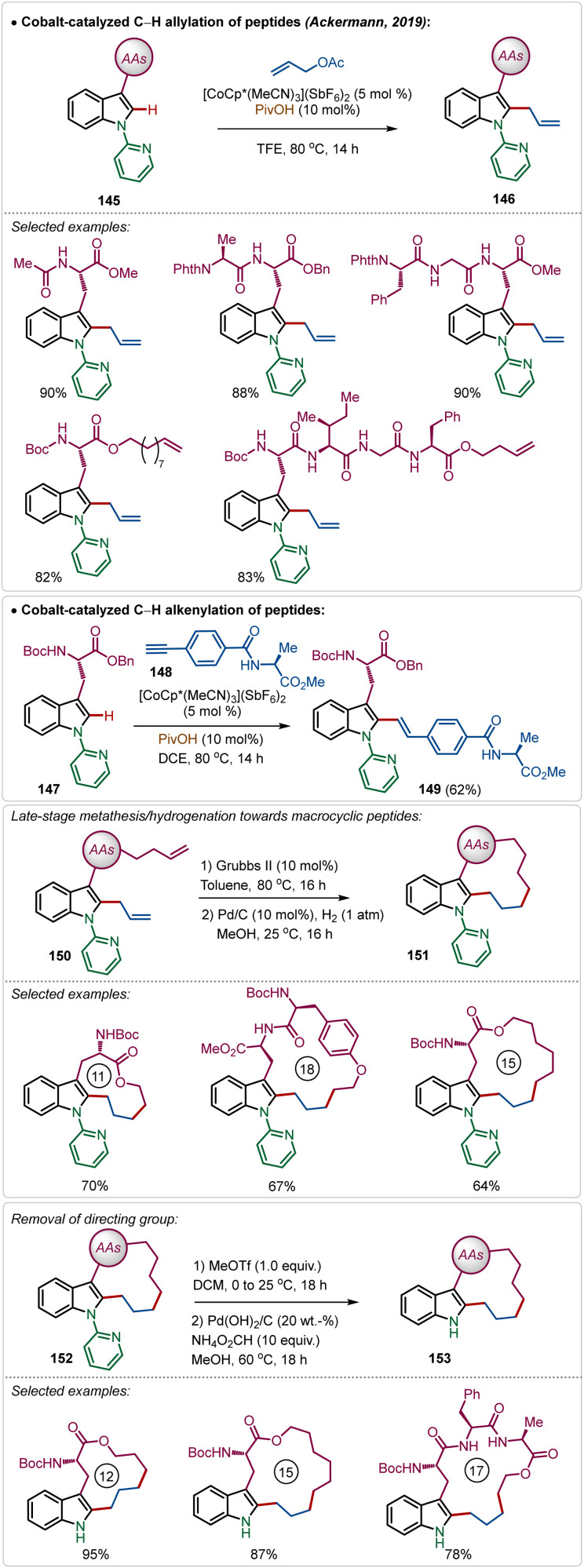
Late-stage functionalization of peptides with Co(iii)-catalysis.

Recently, Ackermann has also explored Ag(i)-additive free Co(iii)-catalyzed protocol for the late-stage modification of peptides (Scheme 26).^77^ Tryptophan-derived peptides were easily allylated with allyl acetate using the electrophilic Co(iii)-catalyst. The reaction conditions were amenable to stitching amino acid derived terminal alkyne generating olefinated products (146). Interestingly, the reaction conditions tolerated olefin functionality in the substrate. Thus, the allylated products were easily manipulated into various cyclic peptides having modular ring sizes.

**Scheme 27:**
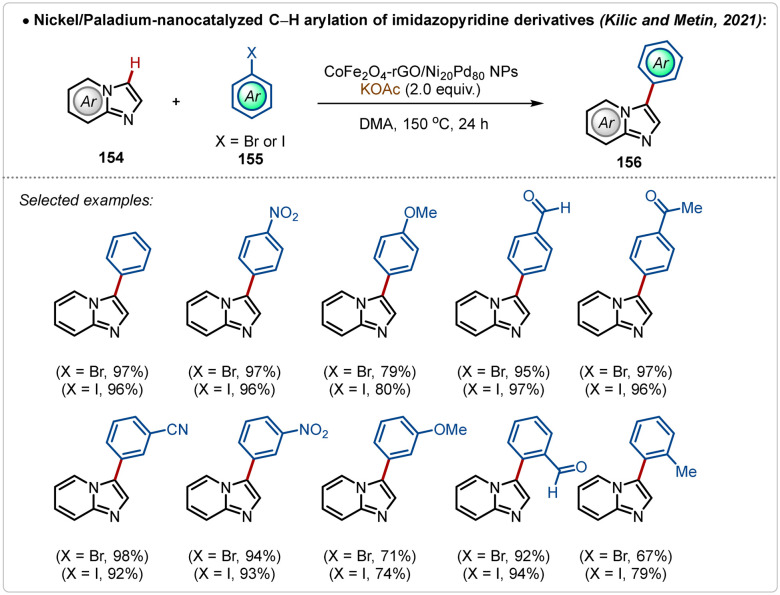
Nickel/paladium-nanocatalyzed Ag-free C–H arylation of imidazopyridine derivatives.

An alternative useful way to avoid silver additives in C–H activation reactions is nanocatalysis.^78^ Such approaches are elusive and require superficial availability of the transition-metal containing nano-catalyst supported on the surface to mediate C–H activation. In 2021, Kilic, Metin and co-workers depicted an example^78*a*^ of C–H arylation of imidazopyridine with bromo or iodobenzene using a Ni-Pd based nanocatalyst (Scheme 27). In this study, a nanocatalyst was designed using a nickel–palladium alloy nanoparticles, supported on a layer of graphene oxide previously modified with cobalt ferrite (CoFe_2_O_4_-rGO/Ni Pd NPs). This modification made it possible to magnetically separate the catalyst from the reaction media and reuse it on five consecutive cycles, without significantly compromising the achieved yield. Using this approach various arylated imidazopyridine derivatives were successfully accessed in good to excellent yields.

## 8. Conclusions

Silver(i)-salts, being one of the most used additives in C–H activation reactions, have been investigated for detailed understanding of their role in different transition-metal catalysis. The preceding sections briefly elaborated on the crucial role of Ag(i)-salts in the generation of catalytically active species typically from dimeric half sandwiches, acting as a halide scavenger. This type of activation process is the common basis in ruthenium, rhodium, iridium, and cobalt catalysis, which makes it an indispensable additive for such transformations. In Pd(ii)-catalyzed reactions, the Ag(i)-salt can act as a key metal to form bimetallic intermediates, which are decisive for product formation and often necessary to govern the selectivity. Herein, we have summarized representative examples of C–H activations involving Ag(i)-additives, along with a mechanistic analysis. Understanding these insights can influence the impending studies and serve as a guideline for the development of new strategies in and beyond organometallic C–H activation chemistry. Furthermore, the full understanding of the role of silver in C–H activation reactions is a crucial step towards sustainable silver-free approaches towards resource-economic electrochemical approaches. This coherent summary of the role of Ag(i)-additives in C–H activation reactions is expected to guide research in C–H activation to major advances, especially for the development of heterobimetallic catalysis and Ag-free strategies in the future.

## Conflicts of interest

There are no conflicts to declare.

## Supplementary Material

## References

[cit1] Rogge T., Kaplaneris N., Chatani N., Kim J., Chang S., Punji B., Schafer L. L., Musaev D. G., Wencel-Delord J., Roberts C. A., Sarpong R., Wilson Z. E., Brimble M. A., Johansson M. J., Ackermann L. (2021). Nature Rev. Meth. Primers.

[cit2] Guillemard L., Kaplaneris N., Ackermann L., Johansson M. J. (2021). Nat. Rev. Chem..

[cit3] Murali K., Machado L. A., de Carvalho R. L., Pedrosa L. F., Mukherjee R., da Silva Júnior E. N., Maiti D. (2021). Chem. – Eur. J..

[cit4] Xue X.-S., Ji P., Zhou B., Cheng J.-P. (2017). Chem. Rev..

[cit5] Ghosh S., Shilpa S., Athira C., Sunoj R. B. (2022). Topics in Catal..

[cit6] de Carvalho R. L., Dias G. G., Pereira C. L. M., Ghosh P., Maiti D., da Silva Júnior E. N. (2021). J. Braz. Chem. Soc..

[cit7] Jiang H., Sun T. Y. (2021). Molecules.

[cit8] Loup J., Zell D., Oliveira J. C. A., Keil H., Stalke D., Ackermann L. (2017). Angew. Chem., Int. Ed..

[cit9] Anand M., Sunoj R. B., Schaefer H. F. (2014). J. Am. Chem. Soc..

[cit10] Kim. Y. L., Park S., Kim J. H. (2020). Eur. J. Org. Chem..

[cit11] Mondal A., van Gemmeren M. (2022). Angew. Chem., Int. Ed..

[cit12] Murai S., Kakiuchi F., Sekine S., Tanaka Y., Kamatani A., Sonoda M., Chatani N. (1993). Nature.

[cit13] Li Y., Liou Y.-C., Oliveira J. C. A., Ackermann L. (2022). Angew. Chem., Int. Ed..

[cit14] Lin Q., Lin Z., Pan M., Zheng Q., Li H., Chen X., Darcel C., Dixneuf P. H., Li B. (2021). Org. Chem. Front..

[cit15] Okada T., Nobushige K., Satoh T., Miura M. (2016). Org. Lett..

[cit16] Biafora A., Krause T., Hackenberger D., Belitz F., Gooßen L. J. (2016). Angew. Chem., Int. Ed..

[cit17] Hogg A., Wheatley M., Domingo-Legarda P., Carral-Menoyo A., Cottam N., Larrosa I. (2022). J. Am. Chem. Soc..

[cit18] Roudesly F., Oble J., Poli G. (2017). J. Mol. Catal. A Chem..

[cit19] Dias G. G., do Nascimento T. A., de Almeida A. K. A., Bombaça A. C. S., Menna-Barreto R. F. S., Jacob C., da Silva Júnior E. N., Ackermann L. (2019). Eur. J. Org. Chem..

[cit20] Dhawa U., Connon R., Oliveira J. C. A., Steinbock R., Ackermann L. (2021). Org. Lett..

[cit21] Liu Y., Chang F., Jiang Q., Ma Z., Liu C. (2018). Synlett.

[cit22] Bairy G., Nandi A., Manna K., Jana R. (2019). Synthesis.

[cit23] Nareddy P., Jordan F., Szostak M. (2018). Org. Lett..

[cit24] Thirunavukkarasu V. S., Raghuvanshi K., Ackermann L. (2013). Org. Lett..

[cit25] Chowdhury D., Dana S., Mandal A., Baidya M. (2019). Chem. Commun..

[cit26] Bu Q., Rogge T., Kotek V., Ackermann L. (2018). Angew. Chem., Int. Ed..

[cit27] Wang C.-A., Chatani N. (2020). Org. Chem. Front..

[cit28] Yuan Y., Zhu J., Yang Z., Ni S.-F., Huang Q., Ackermann L. (2022). CCS Chem..

[cit29] Hazra S., Hirano K., Miura M. (2019). Asian J. Org. Chem..

[cit30] Zhang L., Zhao J., Mou Q., Teng D., Meng X., Sun B. (2020). Adv. Synth. Catal..

[cit31] Tan E., Nannini L. J., Stoica O., Echavarren A. M. (2021). Org. Lett..

[cit32] Jiang Z. T., Huang J., Zeng Y., Hu F., Xia Y. (2021). Angew. Chem., Int. Ed..

[cit33] Wang L., Qu X., Li Z., Peng W.-M. (2015). Tetrahedron Lett..

[cit34] Stuart D. R., Bertrand-Laperle M., Burgess K. M. N., Fagnou K. (2008). J. Am. Chem. Soc..

[cit35] Rakshit S., Patureau F. W., Glorius F. (2010). J. Am. Chem. Soc..

[cit36] Gong T.-J., Xiao B., Liu Z.-J., Wan J., Xu J., Luo D.-F., Fu Y., Liu L. (2011). Org. Lett..

[cit37] Tsai A. S., Tauchert M. E., Bergman R. G., Ellman J. A. (2011). J. Am. Chem. Soc..

[cit38] Khake S. M., Chatani N. (2021). ACS Catal..

[cit39] Wakikawa T., Sekine D., Murata Y., Bunno Y., Kojima M., Nagashima Y., Tanaka K., Yoshino T., Matsunaga S. (2022). Angew. Chem., Int. Ed..

[cit40] Li Q., Liu R., Wei Y., Shi M. (2021). Adv. Synth. Catal..

[cit41] Yabe R., Ebe Y., Nishimura T. (2021). Synthesis.

[cit42] Ryu J., Kwak J., Shin K., Lee D., Chang S. (2013). J. Am. Chem. Soc..

[cit43] Figg T. M., Park S., Park J., Chang S., Musaev D. G. (2014). Organometallics.

[cit44] Sarkar W., Bhowmik A., Das S., Sulekha A. B., Mishra A., Deb I. (2020). Org. Biomol. Chem..

[cit45] Chen X.-Y., Sorensen E. J. (2018). Chem. Sci..

[cit46] Kona C. N., Nishii Y., Miura M. (2019). Angew. Chem., Int. Ed..

[cit47] Khake S. M., Yamazaki K., Ano Y., Chatani N. (2021). ACS Catal..

[cit48] Kim J., Park S.-W., Baik M.-H., Chang S. (2015). J. Am. Chem. Soc..

[cit49] Kim H., Chang S. (2015). ACS Catal..

[cit50] Pan C., Yang Z., Xiong H., Teng J., Wang Y., Yu J.-T. (2019). Chem. Commun..

[cit51] Park H. S., Fan Z., Zhu R.-Y., Yu J.-Q. (2020). Angew. Chem., Int. Ed..

[cit52] Whitaker D., Burés J., Larossa I. (2016). J. Am. Chem. Soc..

[cit53] Yang Y.-F., Cheng G.-J., Liu P., Leow D., Sun T.-Y., Chen P., Zhang X., Yu J.-Q., Wu Y.-D., Houk K. N. (2014). J. Am. Chem. Soc..

[cit54] Fang L., Saint-Denis T. G., Taylor B. L. H., Ahlquist S., Hong K., Liu S., Han L., Houk K. N., Yu J.-Q. (2017). J. Am. Chem. Soc..

[cit55] Feng W., Wang T., Liu D., Wang X., Dang Y. (2019). ACS Catal..

[cit56] Porey S., Zhang X., Bhowmick S., Singh V. K., Guin S., Paton R. S., Maiti D. (2020). J. Am. Chem. Soc..

[cit57] Tian C., Meyer T. H., Stangier M., Dhawa U., Rauch K., Finger L. H., Ackermann L. (2020). Nat. Protoc..

[cit58] Yao Q.-J., Huang F.-R., Chen J.-H., Zhong M.-Y., Shi B.-F. (2023). Angew. Chem., Int. Ed..

[cit59] Friis S. D., Johansson M. J., Ackermann L. (2020). Nat. Chem..

[cit60] Ozols K., Onodera S., Woźniak Ł., Cramer N. (2021). Angew. Chem., Int. Ed..

[cit61] Sen M., Emayavaramban B., Barsu N., Premkumar J. R., Sundararaju B. (2016). ACS Catal..

[cit62] Sauermann N., González M. J., Ackermann L. (2015). Org. Lett..

[cit63] Ramachandran K., Anbarasan P. (2017). Eur. J. Org. Chem..

[cit64] Liu M., Niu J.-L., Yang D., Song M.-P. (2020). J. Org. Chem..

[cit65] Detmar E., Müller V., Zell D., Ackermann L., Breugst M. (2018). Beilstein J. Org. Chem..

[cit66] Kong L., Biletskyi B., Nuel D., Clavier H. (2018). Org. Chem. Front..

[cit67] Patel P., Chang S. (2015). ACS Catal..

[cit68] Zhu C., Ang N. W. J., Meyer T. H., Qiu Y., Ackermann L. (2021). ACS Cent. Sci..

[cit69] Wang Y., Simon H., Chen X., Lin Z., Chen S., Ackermann L. (2022). Angew. Chem., Int. Ed..

[cit70] Sadowski B., Yuan B., Lin Z., Ackermann L. (2022). Angew. Chem., Int. Ed..

[cit71] Sadowski B., Yuan B., Lin Z., Ackermann L. (2022). Angew. Chem., Int. Ed..

[cit72] Wang X. W., Li X. T., Xiao J., Jiang Y., Li X. W. (2012). Synlett.

[cit73] Nan J., Yin J., Gong X., Hu Y., Ma Y. (2021). Org. Lett..

[cit74] Ikemoto H., Yoshino T., Sakata K., Matsunaga S., Kanai M. (2014). J. Am. Chem. Soc..

[cit75] Fukagawa S., Kato Y., Tanaka R., Kojima M., Yoshino T., Matsunaga S. (2019). Angew. Chem., Int. Ed..

[cit76] Wang H., Lorion M. M., Ackermann L. (2016). Angew. Chem., Int. Ed..

[cit77] Lorion M. M., Kaplaneris N., Son J., Kuniyil R., Ackermann L. (2019). Angew. Chem., Int. Ed..

[cit78] Zozik Y., Sevim M., Lafzi F., Kilic H., Metin Ö. (2021). Dalton Trans..

